# Multimodal Signatures of Brain Aging: From Descriptive Analyses to Machine Learning-Based Integration

**DOI:** 10.3390/ijms27146297

**Published:** 2026-07-15

**Authors:** Chiara Caligiuri, Chiara Feroleto, Marta Morotti, Camilla Codazzi, Federico Frasca, Alessia Cacciotti, Chiara D’Amelio, Federica D’Alelio, Ilaria Paoletti, Lucia Leone, Claudio Grassi, Chiara Pappalettera, Fabrizio Vecchio, Maria Vittoria Podda

**Affiliations:** 1 Department of Neuroscience, Università Cattolica del Sacro Cuore, 00168 Rome, Italy; chiara.caligiuri@unicatt.it (C.C.); chiara.feroleto@unicatt.it (C.F.); camilla.codazzi@unicatt.it (C.C.); chiara.damelio@unicatt.it (C.D.); federica5599@gmail.com (F.D.); lucia.leone@unicatt.it (L.L.); claudio.grassi@unicatt.it (C.G.); mariavittoria.podda@unicatt.it (M.V.P.); 2 Fondazione Policlinico Universitario A. Gemelli IRCCS, 00168 Rome, Italy; marta.morotti@guest.policlinicogemelli.it (M.M.); ila.pao71@gmail.com (I.P.); 3 Brain Connectivity Laboratory, Department of Neuroscience and Neurorehabilitation, IRCCS San Raffaele, 00166 Rome, Italy; federicofrasca14@gmail.com (F.F.); alessiacacciotti1997@gmail.com (A.C.); fabrizio.vecchio@uniecampus.it (F.V.); 4 Department of Theoretical and Applied Sciences, eCampus University, 22060 Novedrate, Italy

**Keywords:** artificial intelligence, brain aging, cognitive decline, dendritic spines, local field potentials, motor dysfunction, EEG, functional connectivity

## Abstract

Understanding age-related neurophysiological changes is crucial for identifying brain aging biomarkers and developing strategies against motor and cognitive decline. To explore aging-related patterns across multiple domains, this study assessed motor and cognitive performance, functional connectivity, and synaptic organization in Young (4 months), Adult (14 months), and Old (24 months) mice. Adult mice exhibited reduced locomotor activity (−50.3%) and forelimb force (−38.3%) compared to Young mice, while Old mice showed decline in spatial (−30.4%) and recognition memory (−40.3%). Golgi–Cox staining revealed region-specific changes in spine density, including an increase in motor cortex layer II/III pyramidal neurons in Old versus Adult mice and reductions in the medial prefrontal cortex and CA1 hippocampal region. Immunofluorescence analysis indicated age-related alterations in VGLUT and VGAT expression across brain regions. Local field potential recordings revealed no significant changes in functional connectivity across age groups. Integration of behavioral and electrophysiological features using machine learning for an exploratory yielded a classification accuracy of 0.798. Although this represented only a modest and non-significant improvement over behavioral features alone, the highest pairwise discrimination was observed between Adult and Old mice (AUC = 0.861). Overall, these findings provide a multilevel descriptive analysis of brain aging, highlighting distinct behavioral and structural alterations alongside preserved functional connectivity.

## 1. Introduction

Aging is a fundamental biological process characterized by progressive functional decline across tissues and organ systems, with the brain being particularly vulnerable to age-related changes [1,2]. Even during physiological aging, in the absence of overt neurodegenerative diseases, individuals commonly experience gradual impairments in motor coordination, learning, memory, and executive function [3,4]. Importantly, aging represents the strongest risk factor for major neurodegenerative disorders, including Alzheimer’s and Parkinson’s diseases, indicating that age-related neural alterations may create a permissive substrate for the emergence of pathology [2]. Therefore, understanding how brain function changes during aging is essential not only for defining the trajectories of healthy aging but also for identifying early alterations that increase the vulnerability to neurodegeneration.

At the cellular level, synaptic integrity is a key determinant of brain function across the lifespan and has been extensively investigated using experimental models that allow direct access to synaptic networks, structures, and plasticity. In particular, dendritic spines, which constitute the primary structural substrate of excitatory synaptic connectivity in cortical and hippocampal circuits, are highly dynamic structures that undergo continuous remodeling in response to experiences and environmental demands [5]. Studies on animal models of brain aging have revealed region- and layer-specific alterations in dendritic spine density and morphology, particularly within circuits supporting motor and cognitive functions [6,7]. However, synaptic aging is not uniform across the brain: while some regions exhibit spine loss, others show preserved or even increased spine density, suggesting that aging involves heterogeneous forms of structural plasticity, the functional significance of which remains incompletely understood [6].

In addition to local synaptic remodeling, aging affects the coordination of neuronal activity across distributed brain networks. Functional connectivity reflects the temporal synchronization of activity between spatially distinct neuronal populations and represents a fundamental organizing principle of brain function that supports efficient information processing and behavior [8]. Age-related alterations in network synchronization have been reported across species, with evidence from human neuroimaging studies and electrophysiological analyses in experimental models, indicating non-linear changes in large-scale network dynamics during aging [9,10,11]. These findings underscore the importance of investigating aging-related processes at the level of large-scale network organization.

In this context, local field potential (LFP) recordings in animal models provide a powerful approach for probing functional brain connectivity, as they predominantly reflect the summed synaptic and dendritic activity of neuronal ensembles with high temporal resolution, bridging cellular-level processes and large-scale network dynamics [9,12,13,14]. LFP-derived connectivity measures offer high signal-to-noise recordings and share strong conceptual similarities with electroencephalography (EEG)-based metrics widely used in humans, highlighting their translational relevance in investigating brain dynamics across the lifespan. However, because aging-related alterations in functional connectivity are often subtle, distributed, and follow non-linear trajectories, their functional relevance may not be fully captured by conventional univariate analytical approaches alone [10,11].

Behavioral phenotypes reflect the integrated output of neural systems and therefore provide functional readouts of age-dependent changes at the molecular and structural levels that complement synaptic and network-level measures [15]. In this framework, machine learning approaches provide a powerful framework for integrating multidimensional datasets and identifying multivariate aging signatures that extend beyond single-domain analyses [16]. Such integrative approaches are particularly suited to the study of physiological aging, where alterations may be modest at the individual level but emerge clearly when considered in combination.

In the present study, we investigated age-related changes in motor and cognitive behaviors, dendritic spine density, and markers of excitatory and inhibitory pre-synaptic terminals across cortical and hippocampal regions, which were selected based on their established sensitivity to aging-related alterations at distinct levels of brain organization. Motor and cognitive performance serve as behavioral readouts of cumulative neural decline [17]; dendritic spine density and pre-synaptic markers capture structural and functional plasticity at the synaptic level, where region-specific remodeling represents a cardinal feature of cortical and hippocampal aging [18]. In parallel, we assessed large-scale functional coupling using total coherence (TotCoh) derived from LFP recordings in mice spanning young to old age, which probed network-level dynamics constituting a distinct level of brain organization not directly predictable from local synaptic organization alone [19]. By integrating behavioral measures with LFP-derived TotCoh across multiple frequency bands using supervised machine learning and feature selection approaches, we tried to identify the multimodal signatures of physiological brain aging. This approach could allow us to examine how behavioral, synaptic, and network-level alterations jointly could contribute to aging-related functional phenotypes, with implications for possible understanding of vulnerability to neurodegenerative diseases.

## 2. Results

### 2.1. Behavioral Assessments

**Figure 1:**
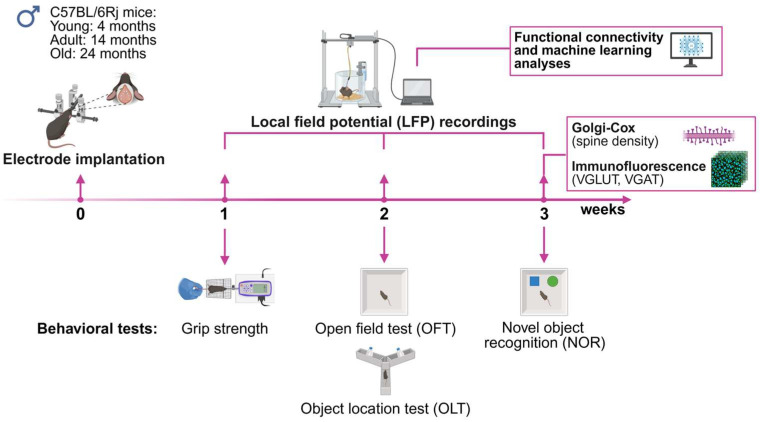
**Experimental timeline.** All mice underwent electrode implantation (time 0), with local field potential (LFP) recordings taken at 1, 2, and 3 weeks post-surgery. Behavioral assessments included grip strength at 1 week, open field test (OFT) and object location test (OLT) at 2 weeks, and novel object recognition (NOR) at 3 weeks after surgery. After the last testing session, the mice were sacrificed, and their brains were collected for Golgi-Cox staining and immunofluorescence analyses for VGLUT and VGAT. The remaining tissues were stored for further analysis. LFP recordings were used for functional connectivity and machine learning analyses. The figure was created in BioRender.

C57BL/6JRj male mice were divided into three age groups (Young [4-month-old], Adult [14-month-old] and Old [24-month-old]) and were tested for motor and cognitive performance using a battery of validated behavioral tests, which were administered over a 3-week period, starting from 1 week after electrode implantation for LFP recordings. In particular, the grip strength test was used to assess forelimb strength at week 1, the open field test (OFT) and the object location test (OLT) were used to evaluate general locomotor activity and spatial memory, respectively, at week 2, and the novel object recognition (NOR) test was used to assess long-term recognition memory at week 3 after electrode implantation. After the last testing session, the mice were sacrificed, and their brain tissues were collected. A subset of the samples was processed for spine density and pre-synaptic marker analyses (Figure 1; see Section 4 for details on sample size, experimental design, and exclusion/inclusion criteria), whereas the remaining tissues were stored for future analyses.

#### 2.1.1. Motor Deficits Emerge in Adulthood and Persist in Old Age

**Figure 2:**
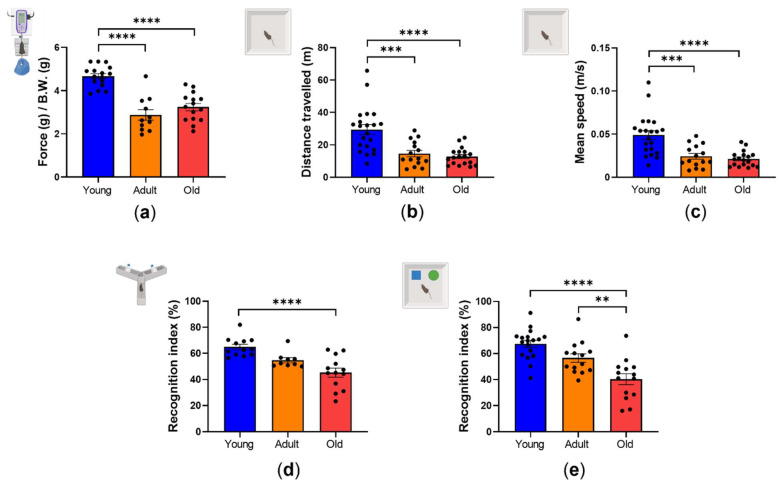
**Behavioral assessment of motor and cognitive functions in Young, Adult and Old mice.** (**a**) Grip strength test performed 1 week post-surgery revealed reduced forelimb force in Adult (*n* = 11) and Old mice (*n* = 15) compared with Young group (*n* = 16 mice). (**b**,**c**) OFT carried out 2 weeks post-surgery showed decreased locomotor activity in Adult (*n* = 15) and Old mice (*n* = 18) compared with Young mice (*n* = 21) (**b**) and decreased mean speed (**c**) in Adult (*n* = 15) and Old mice (*n* = 18) compared with Young group (*n* = 21 mice). (**d**) OLT performed 2 weeks post-surgery demonstrated impaired spatial memory in Old mice (*n* = 13) compared with the Young group (*n* = 12 mice). No statistically significant difference was observed between Adult (*n* = 9) and Young mice. (**e**) NOR conducted 3 weeks post-surgery revealed a decline in recognition memory in Old mice (*n* = 14) compared with both Young (*n* = 18) and Adult (*n* = 14) mice. Data are presented as mean ± standard error of the mean (SEM). ** *p* < 0.01, *** *p* < 0.001, **** *p* < 0.0001; one-way ANOVA, Bonferroni post hoc. Each dot represents an individual animal. B.W., body weight. The figure was created in BioRender.

Statistical analysis revealed reduced forelimb force (force [g]/body weight [g]) in both Adult and Old mice compared with the Young group (Adult vs. Young: 2.88 ± 0.24 vs. 4.67 ± 0.12; *p* < 0.0001; one-way ANOVA, Bonferroni post hoc; Cohen’s d = 2.86; 95% Confidence interval, CI: −2.43, −1.16; Figure 2a; Old vs. Young: 3.24 ± 0.17 vs. 4.67 ± 0.12; *p* < 0.0001; one-way ANOVA, Bonferroni post hoc; Cohen’s d = 2.50; 95% CI: −2.01, −0.85; F_(2,39)_ = 30.98; *p* < 0.0001; Young: *n* = 16 mice; Adult: *n* = 11 mice; Old: *n* = 15 mice; Figure 2a). No difference was observed between the Adult and Old groups (2.88 ± 0.24 vs. 3.24 ± 0.17; *p* = 0.486; one-way ANOVA, Bonferroni post hoc; Figure 2a). These findings suggest that a decline in forelimb strength appears during adulthood and remains relatively stable thereafter.

General locomotor activity was assessed using the OFT. The results showed that both Adult and Old mice exhibited reduced locomotor activity compared to the Young group as indicated by a significant decrease in total distance traveled (Adult [*n* = 15 mice] vs. Young [*n* = 21 mice]: 14.63 ± 1.98 m vs. 29.41 ± 3.06 m; *p* < 0.001; one-way ANOVA, Bonferroni post hoc; Cohen’s d = 1.25; 95% CI: −23.27, −6.31; Figure 2b; Old [*n* = 18 mice] vs. Young: 12.78 ± 1.26 m vs. 29.41 ± 3.06 m; *p* < 0.0001; one-way ANOVA, Bonferroni post hoc; Cohen’s d = 1.52; 95% CI: −24.69, −8.57; F_(2,51)_ = 15.71; *p* < 0.0001; Figure 2b) and in the mean speed (Adult vs. Young: 0.03 ± 0.003 m/s vs. 0.05 ± 0.01 m/s; *p* = 0.001; one-way ANOVA, Bonferroni post hoc; Cohen’s d = 0.56; 95% CI: −0.04, −0.01; Figure 2c; Old vs. Young: 0.02 ± 0.002 m/s vs. 0.05 ± 0.01 m/s; *p* < 0.0001; one-way ANOVA, Bonferroni post hoc; Cohen’s d = 0.88; 95% CI: −0.04, −0.01; F_(2,51)_ = 13.50; *p* < 0.0001; Figure 2c). No differences were observed between Adult and Old mice in either the distance traveled (14.63 ± 1.98 m vs. 12.78 ± 1.26 m; *p* > 0.999; one-way ANOVA, Bonferroni post hoc; Figure 2b) or in the mean speed (0.03 ± 0.003 m/s vs. 0.02 ± 0.002 m/s; *p* > 0.999; one-way ANOVA, Bonferroni post hoc; Figure 2c). These results indicate that impairments in locomotor performance emerge during adulthood and persist in old age without any additional decline. As age-related behavioral changes encompass motor and higher-order cognitive domains, we assessed recognition and spatial memory across age groups.

#### 2.1.2. Cognitive Deficits Emerge in Old Age

OLT was performed to assess the long-term spatial memory. Old mice displayed a significantly lower recognition index than Young mice (45.20 ± 3.48% vs. 64.93 ± 2.10%; *p* < 0.0001; one-way ANOVA, Bonferroni post hoc; Cohen’s d = 1.90; 95% CI: −29.29, −10.18; F_(2,31)_ = 13.66; *p* < 0.0001; Young: *n* = 12 mice; Adult: *n* = 9 mice; Old: *n* = 13 mice; Figure 2d). No statistically significant differences in the recognition index were observed between Adult and Young mice (54.78 ± 2.01% vs. 64.93 ± 2.10%; *p* = 0.062; one-way ANOVA, Bonferroni post hoc; Figure 2d) or between Adult and Old mice (54.78 ± 2.01% vs. 45.20 ± 3.48%; *p* = 0.077; one-way ANOVA, Bonferroni post hoc; Figure 2d). These results indicate that spatial memory declines with age, with significant impairment in Old mice.

To evaluate long-term recognition memory during aging, we tested mice in the NOR test. The analysis revealed that Old mice showed a deficit in recognition memory as indicated by a lower recognition index compared to both Young and Adult groups (Old vs. Young: 40.29 ± 4.18% vs. 67.44 ± 2.72%; *p* < 0.0001; one-way ANOVA, Bonferroni post hoc; Cohen’s d = 2.02; 95% CI: −38.76, −15.54; Old vs. Adult: 40.29 ± 4.18% vs. 56.53 ± 3.25%; *p* < 0.01; one-way ANOVA, Bonferroni post hoc; Cohen’s d = 1.16; 95% CI: −28.54, −3.92; F_(2,43)_ = 16.99; *p* < 0.0001; Young: *n* = 18 mice; Adult: *n* = 14 mice; Old: *n* = 14 mice; Figure 2e). No statistically significant difference was observed between Adult and Young mice (56.53 ± 3.25% vs. 67.44 ± 2.72%; *p* = 0.06; one-way ANOVA, Bonferroni post hoc; Figure 2e). These results suggest an age-related impairment in recognition memory, which becomes significant in old age.

#### 2.1.3. Distinct Age-Related Trajectories in Motor and Cognitive Measures

Regression-based analyses were performed to formally characterize age-dependent behavioral trajectories across motor and cognitive outcomes. Linear and quadratic models were compared using extra sum-of-squares F tests to determine the model that best described the relationship between age and performance.

**Figure 3:**
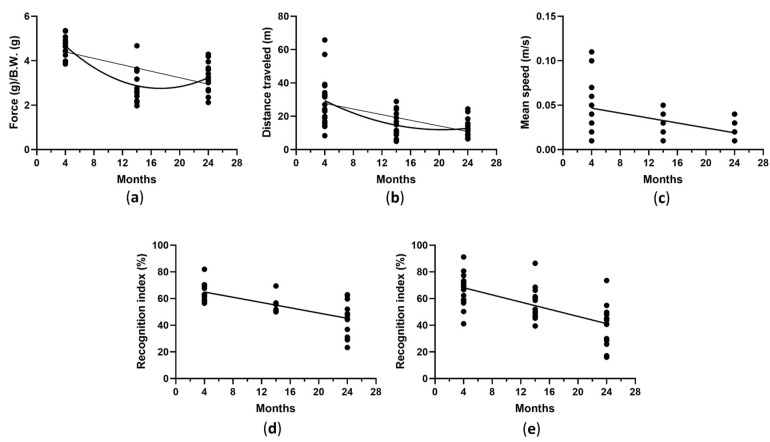
**Age-dependent behavioral trajectories assessed by regression analysis.** (**a**) Quadratic regression of grip strength, revealing a non-linear age-dependent decline. (**b**) Quadratic regression of distance traveled in the Open Field Test, indicating an early locomotor decline followed by partial stabilization at older ages. (**c**) Linear regression of mean speed in the Open Field Test, showing a progressive age-related reduction. (**d**) Linear regression of Object Location Test (OLT) recognition index, showing age-related cognitive decline. (**e**) Linear regression of Novel Object Recognition (NOR) recognition index, showing age-related cognitive decline.

Among motor measures, grip strength exhibited a significant age-related decline in the linear model (R^2^ = 0.388, F_(1,40)_ = 25.34, *p* < 0.0001). However, the quadratic model substantially improved the fit (quadratic R^2^ = 0.613), and model comparison confirmed a significantly better fit than the linear model (F_(1,39)_ = 22.66, *p* < 0.0001), indicating that age-related changes in grip strength follow a non-linear trajectory (Figure 3a).

Similarly, in the Open Field Test, distance traveled decreased significantly with age in the linear model (R^2^ = 0.328, F_(1,52)_ = 25.35, *p* < 0.0001). The quadratic model provided a significantly better fit (R^2^ = 0.381; F_(1,51)_ = 4.410, *p* < 0.05), suggesting a modest non-linear trajectory characterized by an early decline followed by partial stabilization at older ages (Figure 3b). Mean speed also declined significantly with age (R^2^ = 0.304, F_(1,52)_ = 22.74, *p* < 0.0001); however, the quadratic model did not significantly improve the fit (R^2^ = 0.343; F_(1,51)_ = 2.983, *p* = 0.090), indicating that this measure was adequately described by a linear model (Figure 3c).

In contrast, cognitive performance was consistently characterized by linear age-related decline. In the Object Location Test, recognition index declined significantly with age (R^2^ = 0.469, F_(1,31)_ = 27.39, *p* < 0.0001), whereas the quadratic model did not improve model fit (R^2^ = 0.469; F_(1,30)_ = 3.193 × 10^−5^, *p* = 0.996; Figure 3d). Likewise, in the Novel Object Recognition (NOR) test, recognition index declined significantly with age (R^2^ = 0.436, F_(1,44)_ = 34.05, *p* < 0.0001), and the quadratic model failed to provide a better fit (R^2^ = 0.442; F_(1,43)_ = 0.400, *p* = 0.530; Figure 3e). Overall, these analyses revealed distinct age-related behavioral trajectories across domains. Motor-related measures were mostly described by non-linear models, whereas cognitive measures were consistently characterized by progressive linear decline.

### 2.2. Analyses of Dendritic Spine Density and Pre-Synaptic Markers

Given the observed age-dependent functional impairments, spine density and pre-synaptic markers were evaluated using Golgi–Cox staining and immunofluorescence, respectively.

#### 2.2.1. Primary Motor Cortex Exhibits Age-Dependent and Layer-Specific Changes in Dendritic Spine Density

**Figure 4:**
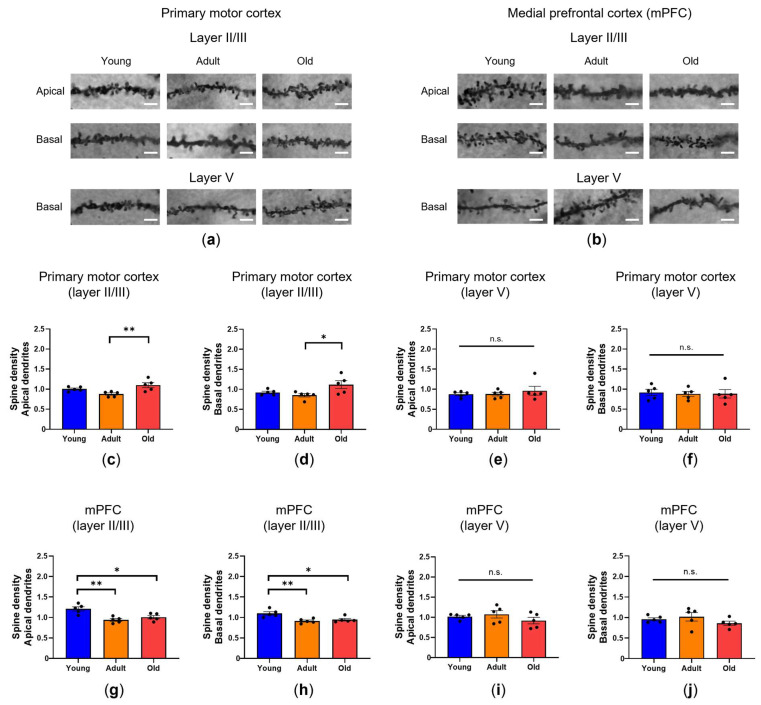
**Dendritic spine density analysis in the primary motor cortex and medial prefrontal cortex.** (**a**,**b**) Representative high-magnification images (100×) of dendritic segments used for spine density analysis in the different experimental groups (*n* = 5 mice/group). Scale bars: 5 µm. (**c**–**f**) In the primary motor cortex, Old mice showed increased spine density in both apical (**c**) and basal (**d**) dendrites of layer II/III pyramidal neurons compared with Adult mice. In layer V, no group differences were observed in apical (**e**) and basal dendrites (**f**). (**g**–**j**) In the medial prefrontal cortex (mPFC), Adult and Old mice exhibited reduced spine density in both apical (**g**) and basal (**h**) dendrites of layer II/III pyramidal neurons compared to Young mice. In layer V, no group differences were observed in apical (**i**) or basal (**j**) dendrites. Data are presented as mean ± SEM. * *p* < 0.05, ** *p* < 0.01; n.s., not significant; one-way ANOVA, Bonferroni post hoc. Each dot represents an individual animal.

One-way ANOVA revealed a significant effect of age on dendritic spine density in both apical (F_(2,12)_ = 6.797, *p* < 0.05; *n* = 5 mice/group; Figure 4a,c) and basal dendrites of pyramidal neurons in layer II/III of the motor cortex (F_(2,12)_ = 4.543, *p* < 0.05; *n* = 5 mice/group; Figure 4a,d). Post hoc comparisons revealed significant differences between Adult and Old mice, with increased spine density in Old mice in both apical (1.10 ± 0.06 vs. 0.88 ± 0.03 spines/µm; *p* < 0.01; Bonferroni post hoc; Cohen’s d = 2.07; 95% CI: −0.39, −0.05; Figure 4c) and basal dendrites (1.12 ± 0.10 vs. 0.85 ± 0.04 spines/μm; *p* < 0.05; Bonferroni post hoc; Cohen’s d = 1.59; CI: −0.52, −0.01; Figure 4d). No significant differences were observed among the groups in layer V pyramidal neurons in apical (Young: 0.88 ± 0.03 spines/μm; Adult: 0.87 ± 0.046 spines/μm; Old: 0.96 ± 0.11 spines/μm; F_(2,12)_ = 0.46; *p* = 0.641; one-way ANOVA; Figure 4e) and basal dendrites (Young: 0.92 ± 0.08 spines/μm; Adult: 0.88 ± 0.06 spines/μm; Old: 0.88 ± 0.11 spines/μm; F_(2,12)_ = 0.058; *p* = 0.944; one-way ANOVA; Figure 4a,f).

#### 2.2.2. Medial Prefrontal Cortex Exhibits Layer-Specific Age-Related Reductions in Dendritic Spine Density

Considering the role of the mPFC in the processing of high-order motor and cognitive functions, dendritic spine density was evaluated in this region. Dendritic spine density was quantified in individual neurons (10–15 per mouse), and values were averaged for each animal (*n* = 5 mice/group) for statistical analysis. One-way ANOVA showed a significant effect of age on both apical (F_(2,12)_ = 11.24, *p* < 0.01; *n* = 5 mice/group; Figure 4b,g) and basal dendrites of pyramidal neurons in layer II/III (F_(2,12)_ = 9.384, *p* < 0.01; *n* = 5 mice/group; Figure 4b,h). Post hoc analyses revealed significant reduction in apical dendritic spine density of layer II/III pyramidal neurons in Adult (0.94 ± 0.03 spines/μm) and Old mice (1.01 ± 0.04 spines/μm) compared with Young mice (1.21 ± 0.05 spines/μm; Adult vs. Young, *p* < 0.01; Cohen’s d = 2.93; CI: −0.44, −0.11; Old vs. Young, *p* < 0.05; Bonferroni post hoc; Cohen’s d = 1.98; CI: −0.37, −0.04; Figure 4g). No difference was evident between Adult and Old mice (*p* = 0.82, Figure 4g). Similar results were obtained for the basal dendrite compartment (Young: 1.10 ± 0.04 spines/μm; Adult: 0.91 ± 0.03 spines/μm; Old: 0.95 ± 0.03 spines/μm; Adult vs. Young, *p* < 0.01; Cohen’s d = 2.40; CI: −0.32, −0.06; Old vs. Young, *p* < 0.05; Cohen’s d = 1.90; CI: −0.28, −0.03; Adult vs. Old, *p* > 0.999; one-way ANOVA, Bonferroni post hoc; Figure 4h).

No differences among groups emerged in layer V pyramidal neurons in either the apical (Young: 1.02 ± 0.03 spines/μm; Adult: 1.07 ± 0.10 spines/μm; Old: 0.92 ± 0.08 spines/μm; F_(2,12)_ = 0.52; *p* = 0.61; one-way ANOVA; Figure 4i) or basal dendrites (Young: 1.0 ± 0.04 spines/μm; Adult: 1.02 ± 0.10 spines/μm; Old: 0.90 ± 0.05 spines/μm; F_(2,12)_ = 3.31; *p* = 0.07; one-way ANOVA; Figure 4b,j).

In summary, morphological analyses indicate that the mPFC experiences an age-related decline in dendritic spine density, affecting layer II/III but not layer V in both Adult and Old mice compared to the Young group.

#### 2.2.3. Aging Is Associated with Decreased Spine Density in Apical Dendrites of Hippocampal CA1 Subfield

**Figure 5:**
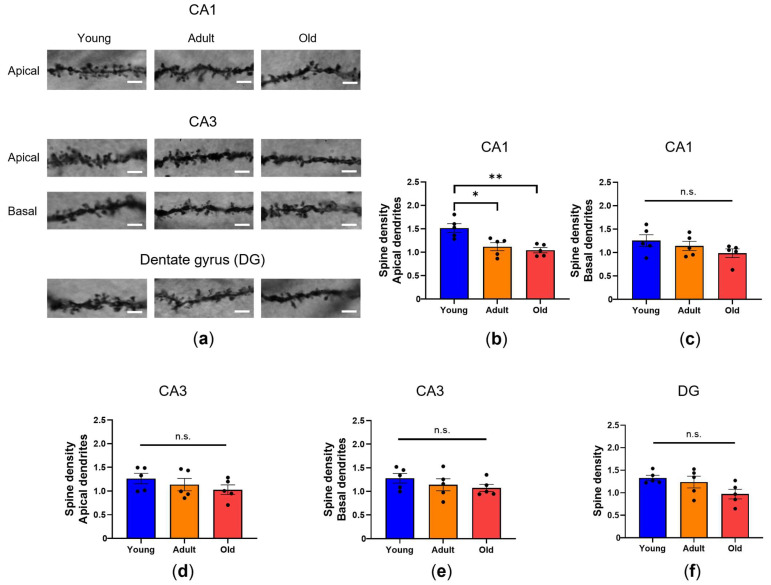
**Characterization of hippocampal dendritic spine density.** (**a**) Representative high-magnification images (100×) of dendritic segments used for spine density analysis in the different experimental groups (*n* = 5 mice/group). Scale bars: 5 µm. (**b**,**c**) In the CA1 region, spine density of apical dendrites (**b**) was reduced in Adult and Old mice compared with Young mice, while no differences were observed in basal dendrites across groups (**c**). (**d**,**e**) In the CA3, no group differences were observed in apical (**d**) and basal dendrites (**e**). (**f**) No group differences were observed in the dentate gyrus (DG). Data are presented as mean ± SEM. * *p* < 0.05, ** *p* < 0.01; n.s., not significant; one-way ANOVA, Bonferroni post hoc. Each dot represents an individual animal.

Dendritic spine density in the CA1, CA3, and dentate gyrus (DG) regions of the hippocampus was also evaluated because behavioral assessments revealed impairments in both object recognition and spatial memory, which are strongly dependent on this region [20]. Dendritic spine density was quantified in individual neurons (10–15 per mouse), and values were averaged for each animal (*n* = 5 mice/group) for statistical analysis. One-way ANOVA showed a significant effect of age on dendritic spine density in the apical dendrites of CA1 pyramidal neurons (F_(2,12)_ = 10.16, *p* < 0.01; *n* = 5 mice/group; Figure 5a,b). Post hoc analyses revealed a significant reduction in Adult (1.12 ± 0.09 spines/μm) and Old mice (1.04 ± 0.06 spines/μm) compared with Young mice (1.52 ± 0.09 spines/μm; Adult vs. Young, *p* < 0.05; Cohen’s d = 1.99; CI: −0.71, −0.08; Old vs. Young, *p* < 0.01; Bonferroni post hoc; Cohen’s d = 2.81; CI: −0.79, −0.16; Figure 5a,b). No difference was evident between Adult and Old mice (*p* > 0.999; Figure 5a,b). Analysis of the basal dendrite compartment revealed no differences among the groups (Young: 1.26 ± 0.13 spines/μm; Adult: 1.14 ± 0.10 spines/μm; Old: 0.99 ± 0.09 spines/μm; F_(2,12)_ = 1.70, *p* = 0.23; one-way ANOVA; Figure 5c). No differences among groups emerged in the CA3 region in either the apical (Young: 1.27 ± 0.11 spines/μm; Adult: 1.14 ± 0.13 spines/μm; Old: 1.03 ± 0.07 spines/μm; F_(2,12)_ = 1.06; *p* = 0.38; one-way ANOVA; Figure 5a,d) or basal dendrites (Young: 1.28 ± 0.10 spines/μm; Adult: 1.14 ± 0.13 spines/μm; Old: 1.07 ± 0.08 spines/μm; F_(2,12)_ = 1.61; *p* = 0.24; one-way ANOVA; Figure 5a,e). The same pattern was observed in the DG (Young: 1.33 ± 0.06 spines/μm; Adult: 1.24 ± 0.13 spines/μm; Old: 0.97 ± 0.11 spines/μm; F_(2,12)_ = 2.81; *p* = 0.10; Figure 5a,f). Taken together, the results of the morphological analyses revealed that age differentially modulates dendritic spine density in motor and cognitive circuits.

#### 2.2.4. Age-Related Differences in Inhibitory and Excitatory Pre-Synaptic Markers Across Neocortical and Hippocampal Regions

**Figure 6:**
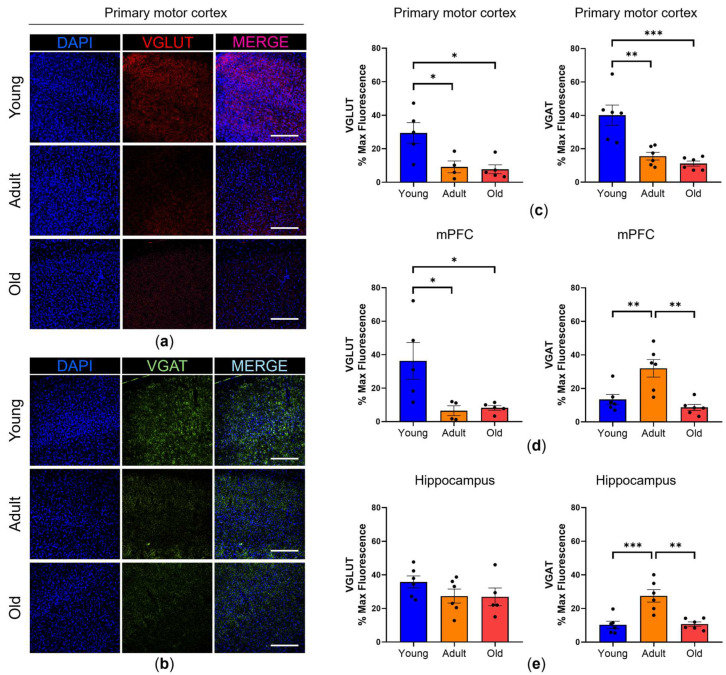
**Immunofluorescence analysis of excitatory and inhibitory pre-synaptic markers across brain regions.** (**a**,**b**) Representative immunofluorescence images showing VGLUT (**a**) and VGAT (**b**) staining with DAPI in the primary motor cortex of Young, Adult, and Old mice. Scale bars: 50 µm. (**c**–**e**) Quantification of VGLUT and VGAT immunofluorescence across age groups in the motor cortex (**c**), mPFC (**d**), and hippocampus (**e**). Data are presented as mean ± SEM. * *p* < 0.05, ** *p* < 0.01, *** *p* < 0.001. Only significant differences are indicated; one-way ANOVA, Bonferroni post hoc. Each dot represents an individual animal (Young: *n* = 5 mice, Adult: *n* = 4 mice, Old: *n* = 5 mice for VGLUT immunofluorescence in primary motor cortex and mPFC; Young: *n* = 6 mice, Adult: *n* = 6 mice; Old: *n* = 5 mice for VGLUT immunofluorescence in the hippocampus; sections from *n* = 6 mice/group were used for VGAT quantification in the different brain areas).

In the primary motor cortex, statistical analysis by one-way ANOVA revealed a main effect of age in the expression of VGLUT (F_(2,11)_ = 7.395, *p* < 0.01) that was significantly lower in Adult (9.20 ± 3.54%; *n* = 4 mice) and Old mice (7.77 ± 2.65%, *n* = 5 mice) than in the Young group (29.40 ± 6.19%; *n* = 5 mice; *p* < 0.05 vs. Adult; Cohen’s d = 1.77; CI: 1.66, 38.75; *p* < 0.05 vs. Old; Bonferroni post hoc; Cohen’s d = 2.03; CI: 4.14, 39.12; Figure 6a,c). No statistically significant differences were found between the Adult and Old groups (*p* > 0.999; Figure 6a,c). A similar age-related effect was observed on VGAT expression (F_(2,15)_ = 16.46, *p* < 0.001; one-way ANOVA). In particular, VGAT immunofluorescence was lower in Adult (15.57 ± 2.31%; *n* = 6 mice) and Old mice (11.14 ± 1.54%; *n* = 6 mice) than in Young mice (40.10 ± 6.05%; *n* = 6 mice; *p* < 0.01 vs. Adult; Cohen’s d = 2.19; CI: 9.88, 39.17; *p* < 0.001 vs. Old; Cohen’s d = 2.68; CI: 14.31, 43.61; Figure 6b,c). The Adult and Old groups did not differ significantly (*p* > 0.999; Figure 6b,c).

In the mPFC, VGLUT immunofluorescence levels differed significantly across groups (F_(2,11)_ = 5.803, *p* < 0.05; one-way ANOVA) with reduced levels in Adult (6.53 ± 2.91%; *n* = 4 mice) and Old animals (8.23 ± 1.38%; *n* = 5 mice) compared to Young mice (36.32 ± 10.96%; *n* = 5; *p* < 0.05 vs. Adult; Cohen’s d = 1.58; CI: 1.03, 58.55; *p* < 0.05 vs. Old; Bonferroni post hoc; Cohen’s d = 1.61; CI: 0.99, 55.21; Figure 6d). The differences between Adult and Old mice were not significant (*p* > 0.999).

A more complex picture emerged with regard to VGAT expression in the mPFC where a main effect of age (F_(2,15)_ = 11.54, *p* < 0.001; one-way ANOVA) was associated with increased VGAT expression in Adult mice (31.99 ± 5.22%; *n* = 6 mice) compared with both Young (13.42 ± 2.99%; *n* = 6 mice; *p* < 0.01; Bonferroni post hoc; Cohen’s d = 1.78; CI: 4.73, 32.4; Figure 5d and Figure S1) and Old animals (8.63 ± 1.82%; *n* = 6 mice; *p* < 0.01; Bonferroni post hoc; Cohen’s d = 0.79; CI: 9.52, 37.19; Figure 6d and Figure S1) while no difference was detected between Young and Old mice (*p* > 0.999; Figure 6d and Figure S1).

Analysis of VGLUT immunofluorescence in the hippocampus did not reveal significant differences among groups (Young: 35.78 ± 3.56%, *n* = 6 mice; Adult: 27.36 ± 4.15%, *n* = 6 mice; Old: 26.90 ± 5.29%, *n* = 5 mice; F_(2,14)_ = 1.392, *p* = 0.2811; one-way ANOVA; Figure 6e). In contrast, VGAT immunofluorescence showed a significant age-dependent modulation (F_(2,15)_ = 14.42, *p* < 0.01; one-way ANOVA). Post hoc analysis revealed a pattern of changes similar to that observed in the mPFC, with higher VGAT levels in Adult mice (27.51 ± 3.70%; *n* = 6 mice) compared with both Young (10.31 ± 2.14%; *n* = 6 mice; *p* < 0.01; Cohen’s d = 2.32; CI: 7.36, 27.04) and Old animals (10.72 ± 1.31%; *n* = 6 mice; *p* < 0.01; Bonferroni post hoc; Cohen’s d = 2.47; CI: 6.94, 26.63), whereas no difference was observed between Young and Old mice (*p* > 0.999; Figure 6e and Figure S1).

Taken together, these findings reveal region- and age-specific differences in inhibitory and excitatory pre-synaptic markers across neocortical and hippocampal regions.

### 2.3. Electrophysiological Measures and Machine Learning

#### 2.3.1. Total Coherence Does Not Significantly Differ Across Age Groups

Because synaptic alterations may influence network-level dynamics, we next assessed age-related changes in large-scale functional coupling by analyzing LFP-derived coherence (TotCoh) across multiple frequency bands, calculated as described in Section 4.9.

Data normality and homoscedasticity were confirmed using the Shapiro–Wilk and Levene tests, respectively (*p* > 0.05).

**Figure 7:**
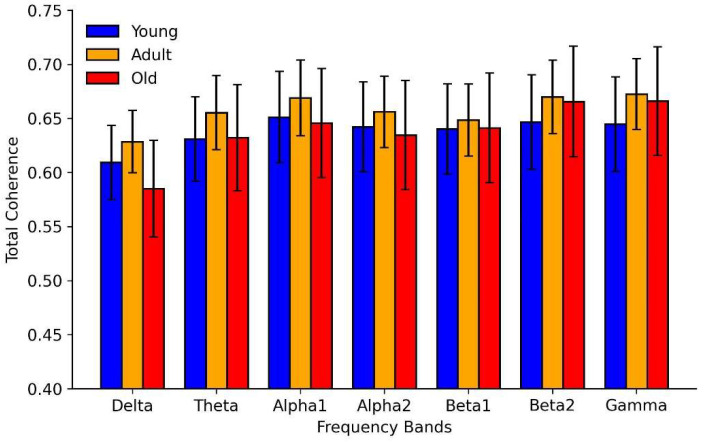
**Total coherence across frequency bands in the Young, Adult, and Old groups.** No statistically significant differences were observed among the age groups (Young: *n* = 21 mice, Adult: *n* = 15 mice; Old: *n* = 18 mice). Data are presented as mean ± SEM. Two-way ANOVA.

To analyze the LFP data, a two-way ANOVA was performed to assess the effect of age on TotCoh values; the analysis revealed no statistically significant main effects or interactions (F_(12, 306)_ = 1.3964, *p* = 0.166). Nevertheless, as shown in Figure 7, a non-significant numerical trend was observed, with the Adult group (*n* = 15 mice) exhibiting consistently higher connectivity across all frequency bands than the Young (*n* = 21 mice) and Old (*n* = 18 mice) groups. This pattern suggests a potential inverted U-shaped trajectory, with a trend toward peak network synchronization during adulthood. However, given the exploratory nature of these findings and the lack of statistical significance, this trajectory should be interpreted as a preliminary observation only.

#### 2.3.2. Age-Group Discrimination Is Enhanced by Multimodal Machine Learning

Given that age-related effects may be subtle and distributed across behavioral and network features, we tested whether multimodal integration improves age-group discrimination using supervised machine learning.

**Figure 8:**
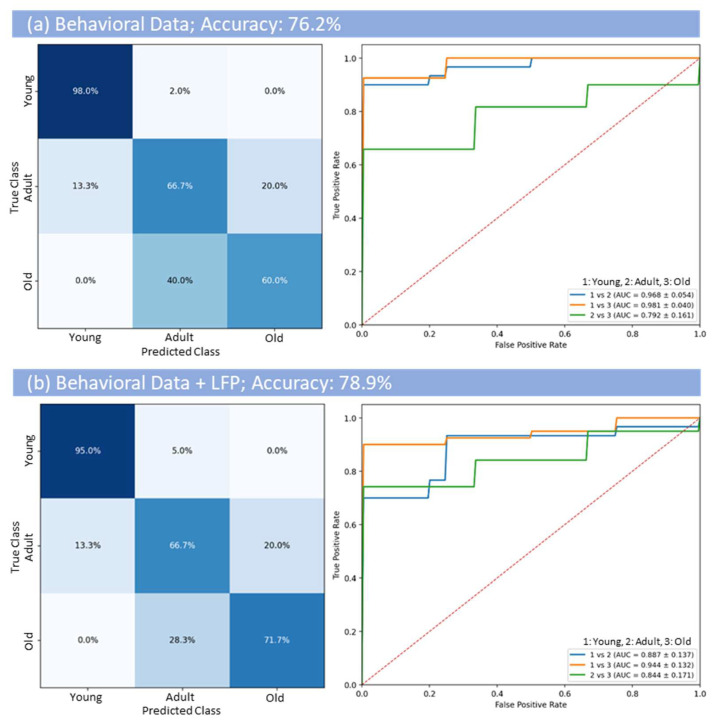
**Classification performance for age-group discrimination based on behavioral features alone (a) and combined behavioral and LFP features (b).** Confusion matrices illustrate the distribution of predicted versus true classes (Young: *n* = 21 mice, Adult: *n* = 15 mice; Old: *n* = 18 mice), with the overall classification accuracy reported for each model. The adjacent matrix shows true-positive rate (TPR) and false-negative rate (FNR) for each age group. Receiver operating characteristic (ROC) curves are reported for the three classes, with dots indicating the selected operating points and corresponding area under the curve (AUC) values.

Machine learning classification based on behavioral features alone (dataset 1) achieved an overall accuracy of 0.762 (95% CI: 0.681–0.842) using a Radial Basis Function Support Vector Classifier (RBF-SVC) (Figure 8a). The model yielded a balanced accuracy of 0.749 (95% CI: 0.665–0.833), a macro-F1 score of 0.730 (95% CI: 0.638–0.822), a macro-sensitivity of 0.749 (95% CI: 0.665–0.833), and a macro-specificity of 0.885 (95% CI: 0.845–0.925). The confusion matrix indicated good discrimination between the Young and Old groups, whereas the Adult group showed a higher rate of misclassification, primarily toward the Old class. Class-wise performance metrics revealed higher true positive rates (TPR) for Young (0.98) and Old (0.60) animals than for Adults (0.67). Receiver operating characteristic (ROC) analysis supported these results, with area under the curve (AUC) values indicating moderate-to-good class separability (Young vs. Adult: 0.968 ± 0.054; Young vs. Old: 0.981 ± 0.040; Adult vs. Old: 0.792 ± 0.161).

When behavioral features were combined with LFP-derived functional connectivity measures (TotCoh across frequency bands; dataset 2), classification performance showed a numerical improvement that did not reach statistical significance, yielding an overall accuracy of 0.788 (95% CI: 0.697–0.880) using a Linear Support Vector Classifier (LinearSVC) (Figure 8b). The model showed a balanced accuracy of 0.778 (95% CI: 0.689–0.867) and a macro-F1 score of 0.759 (95% CI: 0.648–0.869). In addition, the classifier achieved a macro sensitivity of 0.778 (95% CI: 0.689–0.867) and a macro specificity of 0.892 (95% CI: 0.856–0.940). The inclusion of LFP features enhanced class separability, particularly for the Old group, which exhibited a marked increase in TPR (0.72), and for the Young group (TPR = 0.95). Although the true positive rate for the Young group showed a slight decrease, the combined model substantially improved the discrimination between Adult and Old animals, which is an especially challenging distinction in mouse aging studies. Despite the Adult class remaining comparatively less accurate (TPR = 0.67), the model yielded higher AUC values across the comparison between Adult and Old groups (Young vs. Adult: 0.887 ± 0.137; Young vs. Old: 0.994 ± 0.132; Adult vs. Old: 0.844 ± 0.17).

**Figure 9:**
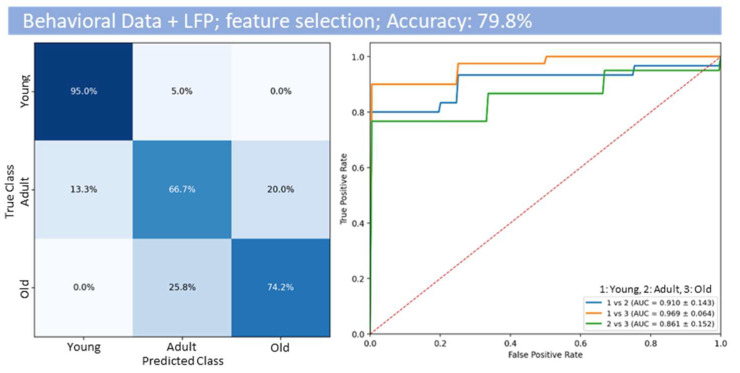
**Classification performance for age-group discrimination using combined behavioral and LFP features after SelectKBest-based feature selection.** The confusion matrix illustrates the distribution of predicted versus true classes (Young: *n* = 21 mice, Adult: *n* = 15 mice; Old: *n* = 18 mice), with an overall classification accuracy derived from the averaged outer test folds. The adjacent matrix reports the class-specific TPR and FNR. The ROC curves for each class, computed via a one-vs.-one strategy, are shown on the right, with dots indicating the selected operating points and corresponding AUC values. Feature selection was embedded in the inner loop to optimize class separability while minimizing information leakage.

Further improvement, although nonsignificant, was achieved using a reduced feature set selected through the feature selection procedure (dataset 3; Figure 9). Using a LinearSVC, the model achieved an overall accuracy of 0.798 (95% CI: 0.725–0.871). The performance metrics showed a balanced accuracy of 0.786 (95% CI: 0.709–0.864) and a macro-F1 score of 0.770 (95% CI: 0.684–0.856). The classifier also achieved a macro sensitivity of 0.786 (95% CI: 0.709–0.864) and a macro specificity of 0.902 (95% CI: 0.871–0.934). Specifically, the selected behavioral features included the grip strength test, NOR, OFT (mean speed and distance traveled), and OLT, whereas the LFP-derived features retained were Delta, Theta, Alpha 1, Alpha 2, and Beta 1 TotCoh. Class-wise true positive rates remained stable for the Young and Adult groups, reaching 0.95 and 0.67, respectively, while the Old group showed a higher TPR (0.74). Additionally, high AUC values were observed across classes (Young vs. Adult: 0.910 ± 0.143; Young vs. Old: 0.969 ± 0.064; Adult vs. Old: 0.861 ± 0.152), indicating good overall discrimination.

## 3. Discussion

Aging of the nervous system does not follow a uniform or linear trajectory; rather, it involves subtle and heterogeneous alterations that progressively impact behavior, synaptic organization, and large-scale network dynamics [21]. Understanding how these changes unfold across multiple levels of brain organization is crucial for elucidating the mechanisms of brain resilience and vulnerability during physiological aging, ultimately guiding the development of targeted strategies to promote healthy brain aging and counteract neurodegeneration. In this context, the present study employed a unified experimental design in which behavioral and electrophysiological measures were obtained in the same cohorts, and synaptic analyses were performed on subsets from these cohorts, enabling the parallel assessment of age-related alterations across domains.

Our findings support a multilevel model of physiological brain aging in which motor, cognitive, synaptic, and network-level domains follow temporally distinct and partially dissociable trajectories.

**Figure 10:**
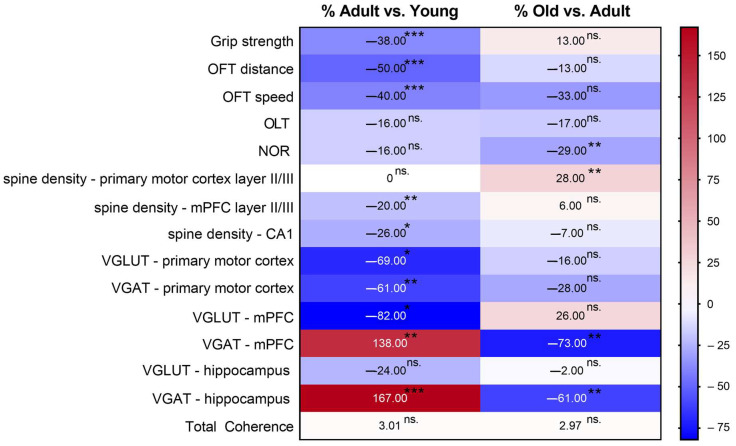
**Heatmap summarizing age-related changes in behavioral, synaptic, and functional parameters.** The heatmap displays the magnitude and direction of changes across age groups, expressed as percentage variation. Values represent relative changes between Adult and Young (% Adult vs. Young) and between Old and Adult (% Old vs. Adult), calculated relative to the mean value of the reference group according to the formula [(Group_2_ − Group_1_)/Group_1_] × 100. Negative values (blue scale) indicate a decrease, whereas positive values (red scale) indicate an increase. Statistical significance determined by one-way ANOVA; Bonferroni post hoc is indicated by asterisks (* *p* < 0.05, ** *p* < 0.01, *** *p* < 0.001 as reported in Section 2). Comparisons that did not reach statistical significance are labeled as ns (i.e., not significant). This descriptive heatmap provides a visual summary of age-related changes across behavioral performance, dendritic spine density, synaptic markers, and large-scale functional connectivity. It is intended to facilitate comparison of the direction and magnitude of changes across domains, rather than to provide an independent analytical or causal framework.

Together, our findings suggest that motor-related systems may exhibit earlier vulnerability to aging than cognitive systems under physiological conditions (Figure 10). This highlights the importance of focusing on the early changes that occur during adulthood. Notably, our study utilized a multivariate approach integrating behavioral and functional connectivity data.

In particular, our behavioral analyses revealed an early decline in motor performance, with reduced forelimb strength and locomotor activity appearing in adulthood and continuing into old age. These findings align with previous lifespan studies indicating that motor decline begins relatively early [17,22]. Importantly, no further decline was observed between Adult and Old animals, suggesting that motor impairments plateau after the initial adult onset rather than progressively worsening.

The regression-based analyses further supported this interpretation by demonstrating that the majority of motor measures were better described by non-linear trajectories, whereas cognitive outcomes followed linear age-dependent decline. These analyses formally demonstrate that age-related behavioral decline does not follow a uniform trajectory across functional domains.

The translational relevance of the early identification of motor dysfunction has been highlighted by clinical studies indicating that impairments in motor skills often occur before cognitive decline in patients with Alzheimer’s disease [23,24].

Cognitive performance, typically assessed in clinical settings among older adults at risk of neurodegeneration, followed a delayed trajectory. Recognition memory, assessed using the OLT and NOR paradigms with a 24 h retention interval, was largely preserved in Adult mice, whereas Old animals exhibited marked impairment, with recognition indices approaching chance level across tasks. Because all animals met stringent exploration criteria during the test phase and displayed preserved locomotor engagement in the open field test, these deficits are unlikely to reflect nonspecific changes in activity or motivation in the animals. Rather, they point to a reduced ability to retain or retrieve object- and spatial-related information over extended delays. Taken together, these findings indicate that cognitive aging in this model is characterized by a relative preservation of recognition memory through adulthood, followed by a more pronounced decline in old age. The convergence of deficits across NOR and OLT suggests that multiple recognition memory domains become vulnerable under conditions of increased memory load, consistent with previous reports [25].

Although causal relationships cannot be directly inferred from the present design, the parallel assessment of behavioral, synaptic, and electrophysiological measures revealed coordinated but partially dissociable age-dependent alterations across domains. In the primary motor cortex, significant differences were observed only between Adult and Old mice, with Old animals showing increased spine density in the apical and basal dendrites of layer II/III pyramidal neurons. We cannot determine from the present data whether these changes reflect adaptive or compensatory mechanisms. In this context, previous studies using longitudinal in vivo imaging have reported age-related increases in spine density and turnover in the motor cortex, suggesting structural remodeling during aging [26].

In the mPFC, we observed layer-specific age-related changes, with a significant reduction in dendritic spine density in both apical and basal compartments of layer II/III neurons in Adult and Old mice compared with Young animals. These findings are consistent with previous studies reporting that age-related structural alterations in prefrontal and hippocampal circuits are associated with cognitive decline in rodents [15,27] and may contribute to the cognitive phenotype observed in older animals. However, based on the present data, we cannot establish a direct link between layer II/III spine alterations and specific behavioral outcomes. It has been proposed that superficial prefrontal layers, which support cortico-cortical integration, may be particularly sensitive to age-related structural changes. In addition, some studies have reported that aging may involve not only spine loss but also altered spine turnover and increased structural stability in the prefrontal cortex, highlighting the complexity of synaptic remodeling during aging [28,29].

Layer V pyramidal neurons did not show significant age-related changes in dendritic spine density in either the mPFC or primary motor cortex, suggesting a relative preservation of cortical output pathways.

Previous studies have reported that aging is associated with structural synaptic alterations in the hippocampus, including reductions in dendritic spine density and changes in synaptic morphology linked to spatial memory performance [7,30].

In our experimental setting, we observed a significant reduction in spine density in the apical dendrites of CA1 pyramidal neurons in both Adult and Old mice compared with Young animals, whereas basal dendrites were relatively preserved. CA1 pyramidal neurons receive major excitatory input from the entorhinal cortex via the temporo-ammonic pathway [31], and apical dendrites may therefore be differentially sensitive to aging-related structural changes. However, based on the present data, we cannot establish a direct relationship between these structural alterations and the behavioral impairments observed in the memory tasks. CA1 is known to play a role in object–context and spatial representations [32,33], although direct mechanistic relationships remain to be established.

Taken together, these observations suggest region- and layer-specific patterns of age-related structural changes across cortical areas. In particular, layer II/III neurons in the primary motor cortex and mPFC, as well as apical dendrites of CA1 pyramidal neurons, are consistent with greater sensitivity to age-related alterations than other compartments examined.

Across regions, we observed different trajectories of spine density changes, with increases in the motor cortex and reductions in the mPFC and hippocampus. These differences suggest that age-related structural alterations are not uniform across brain regions but vary in a region-specific manner.

In parallel, we detected region-specific changes in the pre-synaptic markers of excitatory and inhibitory transmission. However, the present data do not allow us to determine whether post- and pre-synaptic alterations are mechanistically linked or reflect coordinated processes across different brain regions.

Our results are consistent with those of previous studies reporting heterogeneous changes in VGLUT and VGAT expression across cortical and hippocampal circuits during aging [15,34,35]. Particularly noteworthy was the nonmonotonic pattern of VGAT expression observed in both the mPFC and hippocampus, where VGAT levels were increased in Adult mice and returned to lower levels in Old animals. Although the present data do not allow mechanistic conclusions, this transient increase may reflect a compensatory enhancement of GABAergic signaling aimed at maintaining network stability in the presence of early synaptic alterations, including reduced VGLUT expression in the mPFC and age-related changes in dendritic spine density [36]. Such a mechanism could contribute to preserving excitatory–inhibitory balance during adulthood, when cognitive performance remains largely intact. Interestingly, elevated VGAT expression was no longer observed in Old mice, which instead exhibited clear recognition memory deficits. Although speculative, these findings raise the possibility that compensatory inhibitory mechanisms are engaged during adulthood but are no longer maintained during advanced aging. Further studies are required to determine whether this transient VGAT increase represents an adaptive response contributing to cognitive resilience during physiological aging.

To determine whether the region-specific behavioral and synaptic changes identified in this study were accompanied by alterations in large-scale communication, we performed in vivo LFP recordings from multiple brain regions, including the frontal, motor, and parietal cortices, to assess their functional connectivity.

The analysis of LFP-derived total coherence (TotCoh) across various frequency bands did not reveal statistically significant age-related differences in large-scale functional coupling [37]. Adult mice showed slightly and higher coherence values across frequency bands compared with Young and Old animals; however, these differences were not statistically significant and should be considered as exploratory. Therefore, no specific age-related pattern or trajectory of network synchronization can be inferred from the present data, and these observations should be interpreted with caution.

Given the modest and nonsignificant effects observed at the level of individual LFP coherence measures, we employed supervised machine learning to explore whether multimodal integration could enhance age-group discrimination. Classification based on behavioral features alone achieved moderate accuracy, with clearer separation of Young and Old animals but poorer discrimination of Adults. This pattern may reflect the intermediate behavioral profile of adulthood, which shares features with both younger and older stages. Interestingly, the reduced classification performance observed for the Adult group may itself be biologically informative. Behavioral analyses showed that Adult mice already exhibited significant impairments in motor-related measures, including locomotor activity and forelimb strength, whereas cognitive performance remained largely preserved and became significantly impaired only in Old animals. Accordingly, adulthood may represent a transitional stage characterized by the coexistence of early aging-related alterations and preserved cognitive function. Within this framework, the tendency of some Adult animals to be classified as Old may reflect the gradual emergence of aging-associated phenotypes rather than a simple failure of classification, highlighting the non-linear and progressive nature of physiological brain aging. Moreover, the inclusion of LFP-derived functional connectivity measures resulted in a small, non-significant increase in overall classification performance. These findings should be interpreted cautiously, as the electrophysiological measures did not show significant group differences, and no specific contribution to age-related discrimination can be inferred. These findings could indicate that network-level features may provide complementary information to behavioral measures, suggesting that multivariate approaches can capture distributed patterns of change that remain below the detection thresholds of conventional univariate analyses. Feature selection appeared to refine classification while reducing model complexity, indicating that a limited set of non-redundant features may be sufficient to capture the essential aspects of murine brain aging, even if the accuracy gain resulted as nonsignificant. The consistently selected behavioral measures, including motor strength, locomotor activity, and object recognition performance, are well-established readouts of age-dependent functional decline, underscoring the combined contribution of motor and cognitive domains to the aging phenotype, even in this preliminary model [38].

Among the electrophysiological features, TotCoh values in the Delta, Theta, Alpha, and Beta-1 bands were identified as informative predictors within the multimodal machine learning framework. However, it is important to note that the selection of these features by the classifier does not inherently imply direct mechanistic relevance or established biological importance, especially given the absence of significant group-level differences in univariate analyses. Instead, the consistent selection of these frequency ranges may suggest their role as mathematical components of a broader multivariate pattern of change that remains below the detection threshold of conventional statistics. In the murine brain, while these oscillatory bands are known to be involved in hippocampal–cortical communication [39], sensorimotor integration, and cognitive processing [40,41], their contribution to our model should be interpreted with caution as a potential rather than a definitive marker of network-level aging [42]. Within this exploratory framework, the integration of these LFP-derived features with behavioral readouts, such as motor strength (Grip strength test), locomotor activity (OFT), and memory performance (NOR and OLT), provides a preliminary basis for discussing the multivariate signatures often observed in human aging studies [43], without assuming an underlying causal link between feature selection and biological decline.

Together, our findings indicate that behavioral performance, synaptic organization, and network activity are all affected by physiological aging, but they do not necessarily follow a uniform or fully coupled trajectory across the levels of analysis. Instead, each domain shows partially dissociable age-related patterns, suggesting that aging-related alterations emerge in a region- and system-specific manner.

A primary strength of this study is the application of a murine model to integrate behavioral assessments with LFP-derived functional connectivity metrics within a highly controlled environment. This framework establishes a preliminary platform for exploring multivariate aging patterns, offering a level of experimental control over confounding variables that is inherently difficult to achieve in human cohorts.

From a translational perspective, supervised classifiers integrating behavioral and electrophysiological features have been increasingly applied in human aging and neurodegenerative research, where they have been shown to improve age prediction and sensitivity to subtle and distributed network alterations [44,45,46]. However, in humans, the biological interpretation of these multivariate signatures is often limited by interindividual variability and reduced experimental control. By applying a comparable analytical framework in a murine model, our study offers a controlled setting in which the relationship between behavioral and network-level features and age can be investigated. While our results do not establish definitive multivariate “signatures,” they may help inform future efforts to refine and interpret artificial intelligence-based biomarkers of aging in humans.

By applying machine learning after conventional analyses, our approach preserves biological interpretability while enhancing the sensitivity to age-related changes. The present findings suggest that a comprehensive understanding of physiological brain aging may benefit from the parallel assessment of behavioral, synaptic, and network-level measures rather than their isolated evaluation. Overall, the present findings support a model in which physiological brain aging progresses through temporally distinct stages, characterized by early motor-system vulnerability, delayed cognitive decline, region-specific synaptic remodeling, and relatively preserved large-scale network synchronization. Although the integration of multimodal features was associated with a modest increase in classification accuracy, this improvement did not reach statistical significance and should therefore be interpreted with caution. Accordingly, the machine learning results should be considered exploratory, highlighting potential complementary contributions across modalities rather than providing definitive evidence of enhanced classification performance.

This perspective may be relevant, especially when considering the additional variability introduced by sex differences, which have been shown to affect brain aging trajectories and outcomes [47]. In this context, the inclusion of only male animals in the present study represents a limitation, as the generalizability of the findings to female animals is uncertain. Future studies incorporating both sexes are necessary to determine the extent to which the observed age-dependent differences are influenced by sex, thereby improving the interpretability and translational relevance of research findings in this field.

## 4. Materials and Methods

### 4.1. Animals

A total of 54 wild-type C57BL/6JRj male mice were used in this study [48]. The study was designed to maximize statistical power within a feasible sample size; the inclusion of both sexes would have required substantially larger cohorts to account for sex as a biological variable [47]. The mice were housed under standard animal housing conditions with a 12-h light/dark cycle, constant humidity (60–75%), controlled ambient temperature (20–22 °C), and food and water provided ad libitum. All efforts were made to limit the number of animals used and minimize their suffering.

### 4.2. Animal Model and Experimental Design

C57BL/6JRj male mice were divided into three age groups: (i) 4-month-old mice (Young), (ii) 14-month-old mice (Adult), and (iii) 24-month-old mice (Old). Before behavioral testing, all mice underwent a surgical procedure for chronic electrode implantation, followed by LFP recordings performed once a week for three consecutive weeks, starting 1 week after electrode implantation.

The grip strength test was used to assess forelimb strength at week 1, the open field test (OFT) and the object location test (OLT) were used to evaluate general locomotor activity and spatial memory, respectively, at week 2, and the novel object recognition (NOR) test was used to assess long-term recognition memory at week 3 after electrode implantation (Figure 1).

In particular, a pilot cohort of animals (Young: 5 mice; Adult: 4 mice; and Old: 3 mice) underwent the OFT to assess general locomotor activity and the NOR test to assess long-term recognition memory at 2 and 3 weeks after electrode implantation, respectively.

To better explore motor and cognitive functions, the subsequent cohort of animals (Young: 16 mice; Adult: 11 mice; Old: 15 mice), in addition to the OFT and NOR test, were subjected to the grip strength test and to the OLT at 1 and 2 weeks post-electrode implantation, respectively. The total number of animals enrolled in the different tests is summarized in Table 1, which also reports the animals excluded from testing or analyses.

As an internal control, a subset of mice randomly selected from each age group (Young, Adult, and Old; *n* = 5 mice per group) underwent baseline assessment in the NOR test one week prior to surgery to evaluate the potential impact of surgical and experimental procedures on their behavioral performance. The selection of the NOR task was made a priori, based on its sensitivity to age-related cognitive changes, which are most documented in the literature [25]. Within-subject comparisons between baseline and post-surgery measurements (3 weeks post-surgery) did not reveal any statistically significant differences in the recognition index (Figure S2). These data suggest that under the present experimental conditions, the surgical procedure and subsequent experimental handling did not produce detectable changes in NOR performance. However, this control was limited to a subset of animals and a single behavioral domain; therefore, it does not exclude the potential effects on other behavioral or neurobiological measures.

Behavioral assessments were scheduled at different time points across the three-week period to avoid overloading the animals and minimize potential interference between tasks that might affect performance outcomes. Behavioral analyses were performed between 9 a.m. and 4 p.m., at least one day after the recording session. All behavioral tests were conducted after accurately evaluating the health status of the animals. Task-specific inclusion and exclusion criteria were used, as described below.

Data were collected and analyzed in a blinded manner using the ANY-Maze™ behavioral tracking system (Stoelting Co., Wood Dale, IL, USA), except for grip strength measurements, which were scored manually.

At the end of the last behavioral session, all animals were sacrificed, and their brains were collected for subsequent analyses. The experimental design was optimized to minimize the number of animals used while maximizing the data obtained from each mouse. This was achieved by using the same animals for in vivo assessments (i.e., behavioral testing and LFP recordings) and maximizing the use of post-mortem brain tissue across multiple methodological approaches. Specifically, for Golgi–Cox staining, one hemisphere from five animals per group was analyzed, whereas the contralateral hemispheres were used for immunofluorescence analyses. Immunofluorescence was performed on 4–6 animals per group, including one additional hemisphere per group, to complete the dataset. This approach was implemented in accordance with the principles of the 3Rs (Replacement, Reduction, and Refinement).

### 4.3. Inclusion and Exclusion Criteria

For the OFT, animals were excluded from the analysis if they showed a total distance travelled of less than 3 m (excluded *n* = 0 mice). For the NOR and OLT, inclusion criteria required a minimum total exploration time of 5 s during the test phase, with ≥2 s spent on each object and at least three distinct bouts of exploration. These criteria were defined a priori and applied uniformly across all age groups to ensure that discrimination indices were calculated only from animals showing sufficient task engagement during the test session (NOR: excluded *n* = 3 Young, *n* = 1 Adult, *n* = 4 Old mice; OLT: excluded *n* = 2 Adult, *n* = 2 Old mice). In addition, four out of 16 Young mice initially enrolled in the study were not tested in the OLT because their head-mounted electrode assembly loosened after the OFT session and was subsequently secured with additional resin; the mice were not manipulated and were subsequently tested only in the NOR test at week 3 (Table 1). These mice are listed in Table 1 as excluded because of technical problems.

No sensitivity analysis, including animals that did not meet the inclusion criteria, was performed, as under conditions of very low exploration, the resulting recognition indices cannot be considered reliable measures of discrimination. In such cases, small differences in exploration time may generate spuriously high values that do not reflect the true recognition of the novel versus familiar object.

No significant differences in exclusion rates were observed across age groups in the NOR or OLT tasks (two-sided Fisher’s exact test; Table S2), suggesting that exclusions did not introduce a systematic group-specific bias.

### 4.4. Grip Strength Test

Grip strength test was used to assess forelimb force. The mice were suspended by their tails and allowed to grasp a metal grid connected to a force-measuring instrument (GSM, Bioseb Instrument, Vitrolles, France) with their forepaws, and the maximum grip strength was recorded. The forelimb strength values (g) were reported as the average of three consecutive trials normalized to mouse body weight (g) and were used for statistical analysis [13,49,50].

### 4.5. Open Field Test

General locomotor activity was assessed using the OFT. Each mouse was placed at the center of an empty arena (45 × 45 cm) and allowed to explore for 10 min. Throughout the 10 min, the total distance traveled and mean speed were measured using an automated tracking software (ANY-Maze version 7.65). The apparatus was cleaned with a 70% ethanol solution after each session [51].

### 4.6. Novel Object Recognition

The NOR test was performed to assess long-term recognition memory, as previously described [52]. On the first day, the mice were individually subjected to a 10-min habituation session in an empty arena (45 × 45 cm) to familiarize themselves with the apparatus. On the second day, training was conducted in a single 10-min session, in which two identical objects were placed symmetrically in the arena.

On the third day, during the test phase, one of the familiar objects was replaced with a novel object that differed in shape and color, and the mice were allowed to explore for 10 min.

Exploration time (defined as the time the animal snout was directed at the object from <2 cm) was recorded for both novel and familiar objects. The recognition index was calculated as the percentage of time spent exploring the novel object compared to the total object exploration (both novel and old). To further exclude place preference for one side of the arena, the position of the novel object was alternated between both sides during the test session. The objects and arena were cleaned with a 70% ethanol solution before subsequent tests. All objects used for the recognition paradigms were of similar size.

### 4.7. Object Location Test

OLT was used to assess the long-term spatial memory. This test was conducted in a Y-maze apparatus (consisted of three identical arms: length = 35 cm, width = 5 cm, height = 15 cm, arranged at 120° angles from each other, forming a Y), which offers the advantage of enhancing spontaneous exploratory behavior in mice compared to OLT conducted in a square arena, likely due to the increased spatial complexity of the maze itself [25]. The animals were first habituated to the Y-maze apparatus for 10 min without objects. The next day, during the training phase, two identical objects were placed at the end of the two arms of the maze, and the mice were allowed to explore for 10 min. During this phase, visual cues consisting of black triangular cards were placed on the wall next to the objects to facilitate the animals’ spatial orientation. After 24 h, during the test phase, one of the objects was moved to the third previously empty arm, and the animals were allowed to explore for 10 min. The exploration time for each object was then recorded. The recognition index for the displaced object was calculated as the percentage of time spent exploring the displaced object compared with the total object exploration time. To further exclude the place preference for one arm of the maze, the position of the displaced object was alternated between both arms during the test session. The recognition index was calculated as described for the NOR. The Y-maze and objects were cleaned with 70% ethanol between subsequent tests. The objects used were different from those used in the NOR.

### 4.8. Electrode Implantation and LFP Recordings

One week before the start of the behavioral tests, all animals underwent a surgical procedure for chronic electrode implantation, as previously described [13,14]. The mice were anesthetized via an intraperitoneal (i.p.) injection of a mixture of ketamine (87.5 mg/kg) and xylazine (12.5 mg/kg) before being positioned in a stereotaxic frame (Stereotaxic for Mouse, Digital and Portable, SGL M, 68513; RWD, Shenzhen, China). A longitudinal scalp incision was used to expose the skull, and six small burr holes were drilled at stereotaxic coordinates corresponding to the frontal cortex (+2.5 mm anteroposterior and ±0.3 lateral from the Bregma), primary motor cortex (+1.4 mm anteroposterior, ±2 mm lateral from the Bregma) and somatosensory cortex (−0.34 mm anteroposterior, ±2.4 mm lateral from the Bregma) on both hemispheres. Additionally, two more holes were drilled: one for the placement of the reference electrode (+2.5 mm anteroposterior and −2.5 mm lateral from the Bregma) and the other to facilitate the insertion of a skull screw used as a ground (+2.5 mm anteroposterior and +2.5 mm lateral from the Bregma). Eight stainless-steel filaments were soldered to a multipin socket (NPD-18-DD-GS connector, Omnetics, Minneapolis, MN, USA), with six serving as recording electrodes, one serving as the reference, and the last serving as the ground. Each electrode was carefully positioned through the burr holes to ensure electrical contact without damaging the dura mater. This precaution minimizes brain trauma and prevents cerebrospinal fluid leakage. The entire implant was then secured with dental light-curing resin (Tetric Evoflow^®^, Schaan, Liechtenstein), providing a minimally invasive approach to reduce potential intracranial pressure fluctuations caused by cranial exposure to atmospheric conditions. After surgery, the mice were individually housed, allowed 1 week recovery period, and closely monitored for signs of pain or distress. At the end of this period, the animals were individually placed in a recording cage and allowed to move freely during LFP recordings. Each LFP recording session lasted for 30 min. Data acquisition was performed using the Cereplex Direct System (Blackrock Microsystems, Salt Lake City, UT, USA).

### 4.9. LFP Data Analysis

LFP recordings were processed in MATLAB version R2022b (MathWorks, Natick, MA, USA) using custom-developed scripts based on the EEGLAB toolbox version 2024.1

(Swartz Center for Computational Neurosciences, La Jolla, CA, USA). The signals were band-pass-filtered between 0.2 and 47 Hz using a finite impulse response filter. The data were then segmented into 2-s epochs, and segments contaminated by prominent artifacts, including movement-related or environmental noise, were visually inspected and excluded from further analysis. After artifact rejection, at least 23 min of artifact-free data per mouse were retained for subsequent analyses. Functional connectivity analyses were performed in MATLAB using EEGLAB-based routines, as previously described [53]. The coupling between LFP signals was quantified using Magnitude Squared Coherence (MSCoh), which was computed across all possible electrode pair combinations. To provide a global measure of network-level synchronization, coherence values were summarized as the total MSCoh (TotCoh) [54]. Specifically, for each frequency band, the coherence values for each electrode were calculated as the average coherence with all other electrodes, and TotCoh was obtained by averaging these values across electrodes. TotCoh was estimated for each recording and for conventional EEG frequency bands: Delta (2–4 Hz), Theta (4–8 Hz), Alpha 1 (8–10.5 Hz), Alpha 2 (10.5–13 Hz), Beta 1 (13–20 Hz), Beta 2 (20–30 Hz), and Gamma (30–45 Hz) [13,55]. For subsequent analyses, the mean TotCoh value across the three recordings was used.

### 4.10. Golgi–Cox Staining and Dendritic Spine Density Analysis

The brains were processed for Golgi-Cox staining using the FD Rapid GolgiStain™ Kit (FD NeuroTechnologies, Columbia, MD, USA) following the manufacturer’s instructions and published protocols [13,49]. Briefly, the brains were immersed in impregnation solutions, and after 16 days, they were rapidly frozen in liquid nitrogen and stored overnight at −80 °C. Sagittal sections (100 µm) were obtained using a cryostat (Slee MEV Cryostat; Slee Medical GmbH, Nieder-Olm, Germany) at −20 °C. The sections were mounted on gelatin-coated slides, stained, dehydrated, cleared in xylene, and coverslipped with Eukitt^®^ (ORSAtec GmbH, Bobingen, Germany) mounting medium.

Pyramidal neurons in layers II/III and V of the primary motor cortex and mPFC were analyzed in this study. Moreover, pyramidal neurons from the CA1 and CA3 regions, together with granule cells of the DG, were examined in the hippocampus.

Neurons were selected based on the following inclusion criteria: complete and uniform impregnation, absence of background precipitate, planar orientation of dendritic arborization, and relative isolation from neighboring-stained cells. Dendritic spine density was analyzed in the second- and third-order branches of both the apical and basal dendrites. The primary shafts and initial segments (first few micrometers) of each branch were excluded from the analysis because of the typically low spine density in the proximal regions. Quantification of ~20 µm long dendritic segments was performed using Neurolucida 7.5 software (MicroBrightField, Williston, VT, USA) connected to a motorized stage Zeiss microscope equipped with a high-resolution digital camera. Spine density was calculated using Neurolucida Explorer as the number of spines/µm length of the dendritic segment. Spine density was quantified in 10–15 randomly selected neurons per animal and averaged at the animal level, with each animal treated as a single independent data point for statistical analysis (*n* = 5 mice/group).

### 4.11. Immunofluorescence Analyses

Mice were deeply anesthetized (ketamine, 87.5 mg/kg, and xylazine, 12.5 mg/kg, i.p.) and transcardially perfused with cold phosphate-buffered saline (PBS). The brains were extracted, post-fixed in 4% paraformaldehyde at 4 °C for 48 h, and cryoprotected in 30% sucrose in 0.1 M PBS. Sagittal sections (40 µm) were cut using a Leica VT1000S vibratome for immunofluorescence analysis. Free-floating sagittal sections were blocked for 1 h at room temperature (RT; 22–24 °C) in blocking solution (1% BSA, 10% goat serum, 0.5% Triton X-100 in PBS) and incubated overnight at 4 °C with primary antibodies: VGLUT (1:500, Cell Signaling #47181) and VGAT (1:500, Cell Signaling #44498). After washing, the sections were incubated with secondary antibodies (Alexa Fluor 546 donkey anti-rabbit IgG, 1:500, Invitrogen, for VGLUT; Alexa Fluor 488 donkey anti-rabbit IgG, 1:500, Invitrogen, Waltham, MA, USA for VGAT). DAPI was used for nuclear staining [50].

Confocal imaging was performed using a Nikon (Shinagawa, Tokyo, Japan) A1 MP confocal microscope. To minimize acquisition-related bias and ensure comparability across samples, all images were acquired using identical settings (laser power, detector gain, pinhole, pixel dwell time, and z-step), which were kept constant for all experimental groups within each staining experiment. All images were acquired at 20× magnification as Z-stacks spanning a total thickness of 20 µm. Maximum-intensity projections were generated for the quantitative analysis. All images were acquired under non-saturating conditions.

For each animal, two anatomically matched sagittal sections were selected at equivalent rostrocaudal levels based on stereotaxic landmarks. For each section, two non-overlapping fields were acquired in the primary motor cortex and mPFC, whereas four non-overlapping fields were acquired in the hippocampus to sample the CA1, CA2, CA3, and DG regions. Field selection was based on anatomical criteria and was applied consistently across animals to minimize sampling bias.

VGLUT and VGAT immunofluorescence quantification was performed on maximum-intensity projections using ImageJ 1.54p software (NIH, Bethesda, MD, USA). All images for each marker were processed using an identical analysis pipeline to minimize operator bias. Briefly, images were converted to 8-bit and binarized using the Otsu auto-thresholding method implemented in ImageJ, which determines the threshold by minimizing the intra-class intensity variance between the foreground and background pixels. Thresholding was not manually adjusted on an image-by-image basis; instead, consistency was ensured by applying the same automated method and preprocessing steps to all images acquired under the identical conditions. Signal quantification was performed on the resulting binary images to ensure a standardized output independent of manual threshold selection. The entire analysis pipeline (preprocessing, binarization, and quantification) was uniformly applied to all samples. For each animal, the quantified values were averaged across all fields and sections to obtain a single biological replicate, which was used for statistical analysis (*n* = 4–6 mice per group). All image acquisition and analysis procedures were performed blind to experimental group identity.

### 4.12. Artificial Intelligence

Machine learning analyses were performed to classify the mice into three age groups: Young, Adult, and Old, based on three different datasets. The first dataset (dataset 1) comprised the five behavioral variables, the second one (dataset 2) the behavioral variables and TotCoh values across frequency bands, resulting in a total of 12 features. The third dataset (dataset 3) included a reduced set of features obtained through a feature selection technique, described in detail in the following section.

Prior to model training, missing values were handled using a group-wise K-Nearest Neighbors (KNN) imputation procedure. Specifically, this was implemented using the KNeighborsTransformer with n neighbors = 5 and weights = “*distance*”. Missing data were imputed separately within each diagnostic group to preserve group-specific distributions and avoid introducing artificial overlap or information leakage between classes. This approach ensures that missing values are estimated based on the weighted average of the five nearest samples, with closer neighbors contributing more to the imputed value, while continuous variables were maintained as floating-point values to ensure data integrity. Imputation was performed prior to cross-validation using an unsupervised approach applied independently within each group. This choice was motivated by the relatively low proportion and homogeneous distribution of missing values, as well as the need to ensure stable and consistent estimation of multimodal features in a limited sample size. Although this approach may theoretically introduce a risk of data leakage, the imputation procedure did not incorporate any information related to the outcome variable beyond the group stratification.

To ensure rigorous model evaluation and avoid optimistic bias, classification performance was assessed using a nested cross-validation framework. The outer loop was based on a repeated stratified k-fold procedure (5 folds, 2 repeats), ensuring the preservation of class proportions across splits and providing a robust estimate of generalization performance on unseen data. Within each outer training set, an inner 5-fold stratified cross-validation was applied for hyperparameter tuning and model selection, including the optimization of the feature-selection subset.

Four classifiers were compared: Logistic Regression with L2 regularization (liblinear solver), Logistic Regression with L1 regularization (saga solver), LinearSVC, and RBF-SVC. To account for potential class imbalance, all models were trained using class-weight balancing. Hyperparameter optimization was performed through grid search within the inner loop. Specifically, the regularization parameter C was optimized for both logistic regression and support vector models, while the RBF-SVC additionally included optimization of the kernel coefficient *γ*. For the L1-regularized multinomial logistic regression, the parameter grid included C = [0.05, 0.1, 1.0, 10.0]; for L2 logistic regression and LinearSVC, C = [0.1, 1.0, 10.0]; for RBF-SVC, C = [0.1, 1.0, 10.0] and γ = [scale, 0.01, 0.1].

Feature standardization was performed using z-score normalization through StandardScaler, applied within a scikit-learn pipeline to avoid information leakage. Scaling was included for all linear and kernel-based models, and all preprocessing steps were fitted exclusively on the training data of each fold.

Model selection in the inner loop was based on balanced accuracy, which was chosen as the refitting metric to reduce the influence of possible class imbalance. After hyperparameter tuning, the best model from each inner loop was retrained on the full outer training set and then evaluated on the corresponding held-out outer test fold.

For each outer test fold, the following performance metrics were computed: accuracy, balanced accuracy, macro-averaged F1-score, macro-averaged sensitivity (recall), and macro-averaged specificity. Sensitivity and specificity were computed in a one-vs.-rest manner for each class. Specifically, for each class, true positives, false negatives, false positives, and true negatives were derived from the confusion matrix, and class-wise specificity was calculated as TN/(TN + FP). The macro-values were obtained by averaging across classes.

Row-normalized confusion matrices were computed for each outer fold such that each row represented the distribution of predicted labels within each true class. For each model, the normalized confusion matrices were averaged across the outer folds to provide a stable representation of the class-specific classification patterns.

ROC curves and the corresponding AUC values were computed using a dedicated analysis script. In the multiclass setting, ROC analysis was performed using a one-versus-one strategy, generating pairwise ROC curves for each class combination. For each pair of classes, ROC curves were computed within each outer test fold and averaged across folds to obtain a mean ROC curve and corresponding AUC estimates.

The final model performance for each classifier and feature set was summarized as the mean and 95% CI across the outer folds. CI was estimated from the SEM using the t-distribution when available; otherwise, a normal approximation was applied. This procedure enabled a robust comparison of the model performance across alternative feature configurations while minimizing overfitting and information leakage.

All analyses were conducted in Python 3.13 using the libraries NumPy 2.1.0, pandas 2.2.3, scikit-learn 1.6.0, matplotlib 3.10.0, and SciPy 1.14.1, where applicable.

### 4.13. Feature Selection

To reduce the dimensionality and identify the most informative predictors, feature selection was incorporated directly into the machine learning pipeline. Specifically, a univariate filter-based feature selection approach was adopted using SelectKBest 0.18 with the ANOVA F-statistic (f_classif) as scoring function. This method ranks features according to their individual discriminatory power with respect to the multiclass target variable and retains the top k features with the highest scores.

Importantly, feature selection was performed within the nested cross-validation framework, and thus exclusively on the training data of each fold, to avoid information leakage and optimistic bias. In particular, for each model and each outer training fold, the number of selected features (k) was treated as a hyperparameter and optimized within the inner cross-validation loop, together with the classifier-specific hyperparameters. This ensured that feature selection was fully embedded in the model selection procedure and that the test folds remained completely unseen until the final evaluation.

For linear and kernel-based classifiers, the feature selection step was preceded by z-score standardization (StandardScaler) within the same scikit-learn pipeline. Thus, scaling and feature selection were estimated only from the training data in each cross-validation split and then applied to the corresponding validation or test data.

The candidate values for the number of selected features were defined adaptively based on the total number of available predictors. The evaluated values of k included a range of low- to moderate-dimensional solutions (e.g., 2, 3, 4, 5, 6, 8, and 10) as well as the full feature set when appropriate. The optimal number of selected features was determined during the inner-loop grid search, along with the classifier hyperparameters.

To assess the stability of feature selection, a subset of the retained features was recorded for each outer fold and classifier. In addition, a selection frequency analysis was performed by counting the number of outer folds in which each feature was selected. This procedure allowed the identification of the predictors that were most consistently retained across resampling iterations and, therefore, potentially more robustly associated with group discrimination. Features that were consistently selected across the folds were considered stable predictors. In particular, variables selected in at least 80% of the outer cross-validation folds were defined as the most stable features, indicating a consistent contribution to the classification task across independent dataset resampling.

By embedding feature selection within the nested cross-validation procedure, the analysis minimized overfitting and provided a more reliable estimate of the contribution of individual predictors to the classification performance.

### 4.14. Statistical Analysis

The sample size was determined a priori using G*Power 3.1.9.4 software, considering the mean ± standard deviation (SD) of the three groups, based on the results of prior pilot datasets or studies, including our own, using similar methods or paradigms, and assuming a power of 80%, a confidence level of 95%, and a two-tailed type I error rate (α) of 0.05.

Data are expressed as mean ± standard error of the mean (SEM). For all pairwise comparisons, effect sizes (Cohen’s d) were computed using the pooled standard deviation, and the corresponding values for each parameter are provided in the main text and in Table S1. CI are reported for statistically significant biological comparisons in the main text and for machine learning performance metrics derived from nested cross-validation procedures. The data were first tested for normality and equal variance using (Shapiro–Wilk and Levene’s tests). Behavioral and spine density results were analyzed using one-way analysis of variance (ANOVA; factor: groups), followed by Bonferroni post-hoc correction. In addition, regression-based analyses were performed to assess age-dependent behavioral trajectories. Linear and quadratic regression models were fitted for behavioral outcomes, model fit was evaluated using the coefficient of determination (R^2^), and model comparisons were conducted using extra sum-of-squares F tests to determine whether quadratic models significantly improved the fit compared to linear models. Two-sided Fisher’s exact tests were used to compare the proportions of the included versus excluded animals across the experimental groups.

For the analyses of the LFP data, a two-way repeated-measures ANOVA was conducted to examine the effect of age on TotCoh values, considering the factors Group (Young, Adult, Old) and Band (delta, Theta, Alpha 1, Alpha 2, Beta 1, Beta 2, Gamma). The Greenhouse–Geisser correction was applied to account for violations of sphericity. Multiple comparisons were corrected using the Bonferroni correction. Data were analyzed using GraphPad Prism version 8.4.0, SigmaPlot 14.0, or Statistica v.8 (StatSoft Inc., Tulsa, OK, USA).

The unit of statistical analysis was an individual animal in all experiments. All exclusion criteria were defined a priori and applied consistently across groups, as detailed in the Section 4.2. All experiments and analyses were performed in a blinded manner.

## 5. Conclusions

In conclusion, the present findings indicate that physiological brain aging is characterized by nonlinear and domain-specific changes affecting behavior, synaptic structure, and large-scale network activity. Motor impairments emerge early in adulthood and remain relatively stable over time, despite concurrent structural changes in the motor cortical regions. In contrast, cognitive decline becomes more prominent in old age, parallel to synaptic alterations in the prefrontal and hippocampal circuits. No significant age-related differences were detected in the measures of functional connectivity, suggesting preserved large-scale coupling under the present experimental conditions.

In this context, behavioral and electrophysiological features provide complementary information for age group classification. Although exploratory and of modest performance, multivariate approaches may increase sensitivity to distributed age-related changes that are not fully captured by univariate analysis. Notably, while the integration of multimodal features was associated with a modest improvement in classification accuracy, this effect did not reach statistical significance and should therefore be interpreted with caution, supporting an exploratory rather than confirmatory role of machine learning analysis.

Future studies may extend electrophysiological analyses by incorporating additional network-level descriptors, such as entropy-based measures, to better characterize the complexity of functional dynamics during aging. Overall, these findings provide descriptive and exploratory insights into brain aging and support further investigations in larger cohorts using explicit cross-domain analytical approaches.

Limitations: Despite the insights provided by this study, several limitations should be acknowledged. First, the sample size was relatively small and subdivided across age groups, which represents a fundamental constraint relative to the complexity of the machine learning pipeline. Although we employed nested cross-validation to mitigate bias, the high dimensionality of the feature space relative to the number of subjects inherently increased the risk of overfitting and instability in the performance estimates. Furthermore, the small cohort size may lead to instability in the feature selection procedure, meaning that the specific predictors identified by the classifiers should be viewed as illustrative of the multimodal approach rather than as definitive biomarkers. Second, this study lacked an external validation cohort, a common limitation in pilot investigations that restricts the assessment of model robustness in independent datasets. Given that the observed performance gains were modest and did not reach statistical significance, these results must be interpreted strictly as exploratory signal detection within the proof-of-concept framework. Larger-scale investigations with higher subject-to-feature ratios are necessary to validate these multivariate signatures definitively and ensure the stability of the selected features. Additionally, as only male animals were included, the generalizability of these findings to females remains to be determined, particularly considering the potential sex-specific trajectories in brain aging.

## Figures and Tables

**Table 1:** Number of mice included in each behavioral test by age group.

	Test	Initial *n*			Excluded *n*			Final *n*		
		Y	A	O	Y	A	O	Y	A	O
Cohort I	OFT	5	4	3	0	0	0	5	4	3
	NOR	5	4	3	0	0	0	5	4	3
Cohort II	OFT	16	11	15	0	0	0	16	11	15
	NOR	16	11	15	* 3	* 1	* 4	13	10	11
	Grip strength	16	11	15	0	0	0	16	11	15
	OLT	16	11	15	^#^ 4	* 2	* 2	12	9	13

The table reports the initial, excluded, and final numbers (*n*) of mice used in each analysis. Cohort I underwent only the OFT and NOR. Cohort II underwent all four behavioral tests. Y: Young; A: Adult; O: Old. * Mice excluded based on predefined inclusion/exclusion criteria of the test. ^#^ Mice excluded due to technical problems during the test session.

## Data Availability

The original contributions presented in this study are included in the article and Supplementary Materials. Raw data may be shared by the corresponding and senior authors upon reasonable requests.

## Supplementary Materials

The following supporting information can be downloaded at: https://www.mdpi.com/article/10.3390/ijms27146297/s1.

## Abbreviations

The following abbreviations are used in this manuscript:

ANOVA	analysis of variance
AUC	area under the curve
CI	confidence interval
DG	dentate gyrus
EEG	electroencephalography
FNR	false negative rate
KNN	K-Nearest Neighbors
LFP	local field potential
LinearSVC	Linear Support Vector Classifier
mPFC	medial prefrontal cortex
MSCoh	Magnitude Squared Coherence
NOR	novel object recognition
OFT	open field test
OLT	object location test
R^2^	coefficient of determination
RBF-SVC	Radial Basis Function Support Vector Classifier
ROC	receiver operating characteristic
ROI	region of interest
SEM	standard error of the mean
SVC	Support Vector Classifier
TotCoh	Total Coherence
TPR	true positive rate
VGAT	vesicular GABA transporter
VGLUT	vesicular glutamate transporter
