# Supplementary material for: Multimodal Signatures of Brain Aging: From Descriptive Analyses to Machine Learning-Based Integration
Source: Int J Mol Sci. 2026 Jul 15;27(14):6297. doi: 10.3390/ijms27146297 (PMC13410179; doi:10.3390/ijms27146297)
Supplement: Supplementary file 1 [file ijms-27-06297-s001.zip › ijms-4361549-supplementary.pdf]

Supplementary Materials

Supplementary Figure

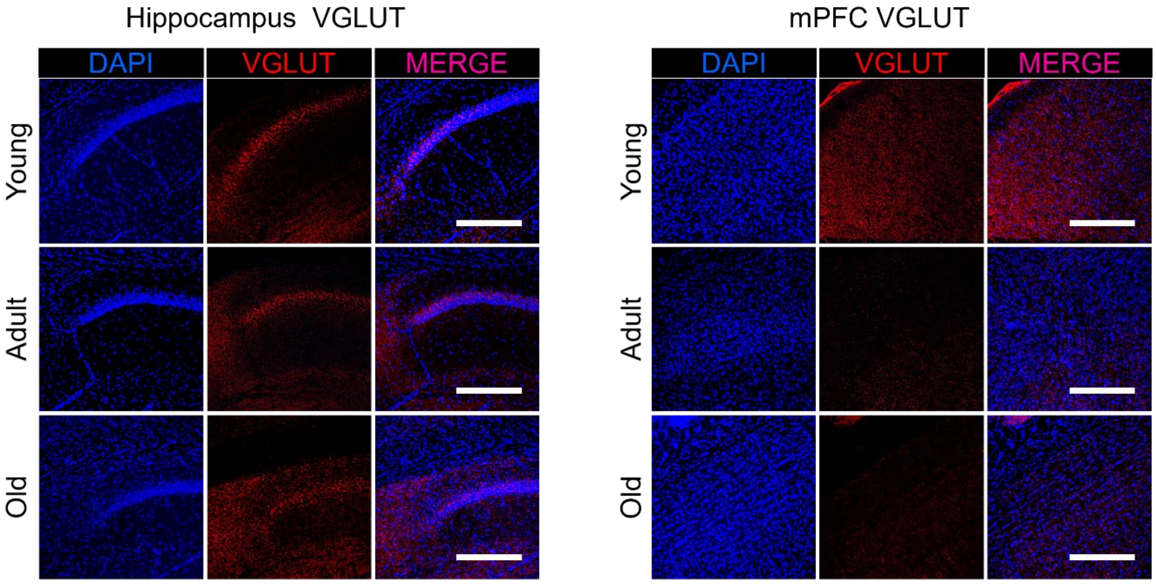

(**a**)

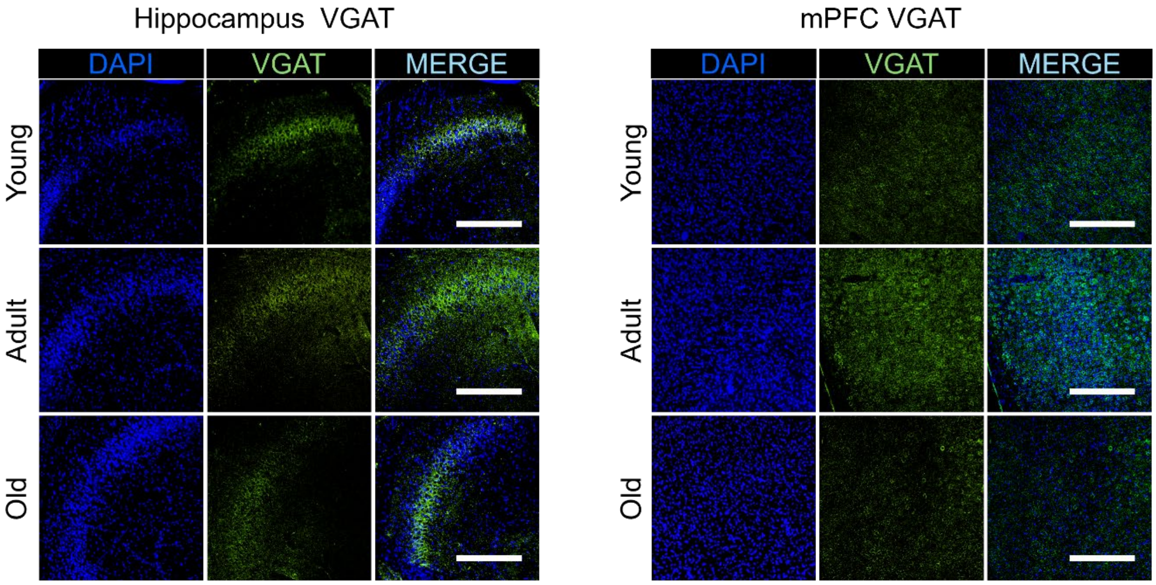

(**b**)

Figure S1. Representative immunofluorescence images of vesicular glutamate transporter (VGLUT) and vesicular GABA transporter (VGAT) staining in the hippocampus and medial prefrontal cortex (mPFC). (**a**) Representative immunofluorescence images showing VGLUT staining with DAPI in the hippocampus and mPFC of Young, Adult, and Old mice. Merged images are shown in the right panels. (**b**) Representative immunofluorescence images showing VGAT staining with DAPI in the hippocampus and mPFC of Young, Adult, and Old mice. Merged images are shown in the right panels. Scale bars: 100 μm.

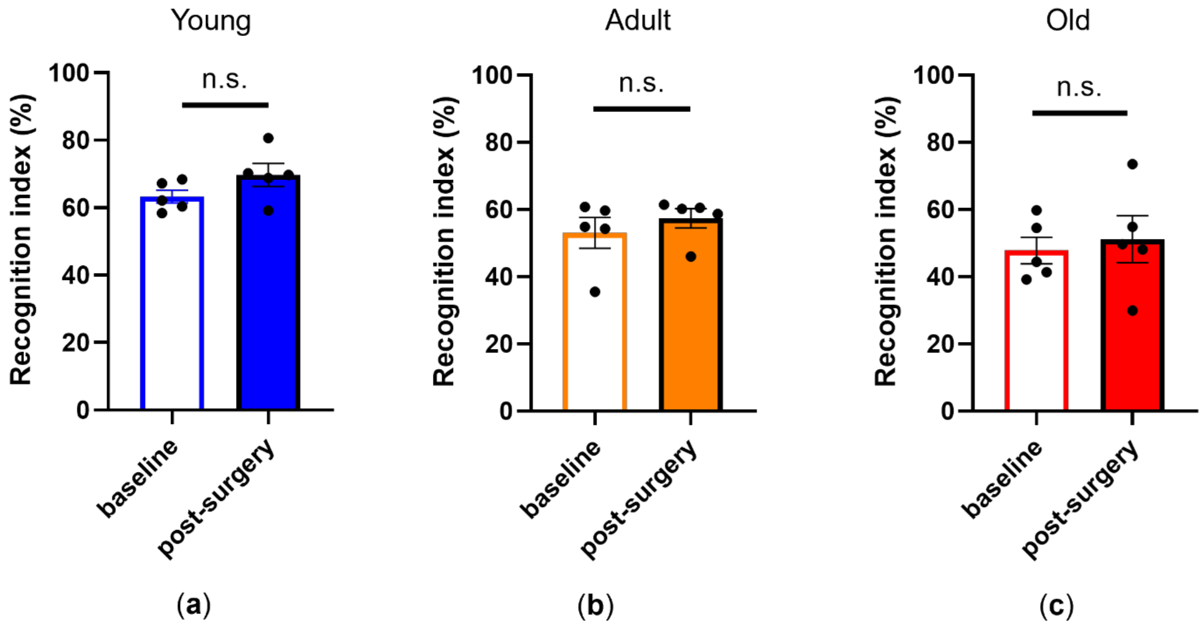

Figure S2. Novel Object Recognition performance before (baseline) and 3 weeks post-surgery in Young (a), Adult (b) and Old mice (c). A subset of mice randomly selected from each age group within the study cohorts underwent baseline assessment in the NOR test one week prior to surgery to evaluate the potential impact of the procedure on behavioral performance. The results indicate no statistically significant differences in the recognition index between baseline measurements and those obtained at the selected post-surgery time point (i.e, 3 weeks post-surgery) in Young (**a**; 63.29±1.94% vs. 69.69±3.40%, *P*=0.25, 95% CI: -6.934 to 19.73; *n*=5), Adult (**b**; 53.04±4.55% vs. 57.40±2.89%, *P*=0.46, 95% CI: -10.49 to 19.23; *n*=5), and Old mice (**c**; 47.86±3.97% vs. 51.23±7.00%, *P*=0.70, 95% CI: -19.59 to 26.34; *n*=5). Data are presented as mean ± standard error of the mean (SEM). Each dot represents an individual animal. Statistical analysis was performed using a paired two-tailed Student's *t*-test. n.s., not significant.

Supplementary Tables

Region / Assay	Endpoint	Young (mean ± SEM; n)	Adult (mean ± SEM; n)	Old (mean ± SEM; n)	ANOVA (F, P)	Computed effect size (Cohen's d)
Grip strength	Forelimb force	4.67 ± 0.12; n=16	2.88 ± 0.24; n=11	3.24 ± 0.17; n=15	F(2,39)=30.98; **P<0.0001**	Adult vs. Young: **2.86**; Old vs. Young: **2.50**; Adult vs. Old: **0.50**
OFT	Total distance traveled	29.41 ± 3.06; n=21	14.63 ± 1.98; n=15	12.78 ± 1.26; n=18	F(2,51)=15.71; **P<0.0001**	Adult vs. Young: **1.25**; Old vs. Young: **1.52**; Adult vs. Old: **0.28**
OFT	Mean speed	0.05 ± 0.01; n=21	0.03 ± 0.00; n=15	0.02 ± 0.00; n=18	F(2,51)=13.50; **P<0.0001**	Adult vs. Young: **0.56**; Old vs. Young: **0.88**; Adult vs. Old: **1.00**
OLT	Recognition index	64.93 ± 2.10; n=12	54.78 ± 2.01; n=9	45.20 ± 3.48; n=13	F(2,31)=13.66; **P<0.0001**	Adult vs. Young: **1.50**; Old vs. Young: **1.90**; Adult vs. Old: **0.92**
NOR	Recognition index	67.44 ± 2.72; n=18	56.53 ± 3.25; n=14	40.29 ± 4.18; n=14	F(2,43)=16.99; **P<0.0001**	Adult vs. Young: **0.92**; Old vs. Young: **2.02**; Adult vs. Old: **1.16**
M1 layer II/III apical	Spine density	n=5	0.88 ± 0.03; n=5	1.10 ± 0.06; n=5	F(2,12)=6.797; **P<0.05**	Adult vs. Old: **2.07**
M1 layer II/III basal	Spine density	n=5	0.85 ± 0.04; n=5	1.12 ± 0.10; n=5	F(2,12)=4.543; **P<0.05**	Adult vs. Old: **1.59**
M1 layer V apical	Spine density	0.88 ± 0.03; n=5	0.87 ± 0.046; n=5	0.96 ± 0.11; n=5	F(2,12)=0.46; **P=0.641**	Adult vs. Young: **0.12**; Old vs. Young: **0.44**; Adult vs. Old: **0.48**
M1 layer V basal	Spine density	0.92 ± 0.08; n=5	0.88 ± 0.06; n=5	0.88 ± 0.11; n=5	F(2,12)=0.058; **P=0.944**	Adult vs. Young: **0.25**; Old vs. Young: **0.19**; Adult vs. Old: **0.00**
mPFC layer II/III apical	Spine density	1.21 ± 0.05; n=5	0.94 ± 0.03; n=5	1.01 ± 0.04; n=5	F(2,12)=11.24; **P<0.01**	Adult vs. Young: **2.93**; Old vs. Young: **1.98**; Adult vs. Old: **0.89**
mPFC layer II/III basal	Spine density	1.10 ± 0.04; n=5	0.91 ± 0.03; n=5	0.95 ± 0.03; n=5	F(2,12)=9.384; **P<0.01**	Adult vs. Young: **2.40**; Old vs. Young: **1.90**; Adult vs. Old: **0.60**
mPFC layer V apical	Spine density	1.02 ± 0.03; n=5	1.07 ± 0.10; n=5	0.92 ± 0.08; n=5	F(2,12)=0.52; **P=0.61**	Adult vs. Young: **0.30**; Old vs. Young: **0.74**; Adult vs. Old: **0.74**
mPFC layer V basal	Spine density	1.00 ± 0.04; n=5	1.02 ± 0.10; n=5	0.90 ± 0.05; n=5	F(2,12)=3.31; **P=0.07**	Adult vs. Young: **0.12**; Old vs. Young: **0.99**; Adult vs. Old: **0.68**
Hippocampus CA1 apical	Spine density	1.52 ± 0.09; n=5	1.12 ± 0.09; n=5	1.04 ± 0.06; n=5	F(2,12)=10.16; **P<0.01**	Adult vs. Young: **1.99**; Old vs. Young: **2.81**; Adult vs. Old: **0.47**
Hippocampus CA1 basal	Spine density	1.26 ± 0.13; n=5	1.14 ± 0.10; n=5	0.99 ± 0.09; n=5	F(2,12)=1.70; **P=0.23**	Adult vs. Young: **0.46**; Old vs. Young: **1.08**; Adult vs. Old: **0.71**

Hippocampus CA3 apical	Spine density	1.27 ± 0.11; n=5	1.14 ± 0.13; n=5	1.03 ± 0.07; n=5	F(2,12)=1.06; **P=0.38**	Adult vs. Young: **0.48**; Old vs. Young: **1.16**; Adult vs. Old: **0.47**
Hippocampus CA3 basal	Spine density	1.28 ± 0.10; n=5	1.14 ± 0.13; n=5	1.07 ± 0.08; n=5	F(2,12)=1.61; **P=0.24**	Adult vs. Young: **0.54**; Old vs. Young: **1.04**; Adult vs. Old: **0.29**
Hippocampus dentate gyrus	Spine density	1.33 ± 0.06; n=5	1.24 ± 0.13; n=5	0.97 ± 0.11; n=5	F(2,12)=2.81; **P=0.10**	Adult vs. Young: **0.40**; Old vs. Young: **1.82**; Adult vs. Old: **1.00**
Cortex	VGLUT	29.40 ± 6.19; n=5	9.20 ± 3.54; n=4	7.77 ± 2.65; n=5	F(2,11)=7.395; **P<0.01**	Adult vs. Young: **1.77**; Old vs. Young: **2.03**; Adult vs. Old: **0.22**
Cortex	VGAT	40.10 ± 6.05; n=6	15.57 ± 2.31; n=6	11.14 ± 1.54; n=6	F(2,15)=16.46; **P<0.001**	Adult vs. Young: **2.19**; Old vs. Young: **2.68**; Adult vs. Old: **0.92**
mPFC	VGLUT	36.32 ± 10.96; n=5	6.53 ± 2.91; n=4	8.23 ± 1.38; n=5	F(2,11)=5.803; **P<0.05**	Adult vs. Young: **1.58**; Old vs. Young: **1.61**; Adult vs. Old: **0.38**
mPFC	VGAT	13.42 ± 2.99; n=6	31.99 ± 5.22; n=6	8.63 ± 1.82; n=6	F(2,15)=11.54; **P<0.001**	Adult vs. Young: **1.78**; Old vs. Young: **0.79**; Adult vs. Old: **2.44**
Hippocampus	VGLUT	35.78 ± 3.56; n=6	27.36 ± 4.15; n=6	26.90 ± 5.29; n=5	F(2,14)=1.392; **P=0.281**	Adult vs. Young: **0.89**; Old vs. Young: **0.87**; Adult vs. Old: **0.04**
Hippocampus	VGAT	10.31 ± 2.14; n=6	27.51 ± 3.70; n=6	10.72 ± 1.31; n=6	F(2,15)=14.42; **P<0.01**	Adult vs. Young: **2.32**; Old vs. Young: **0.09**; Adult vs. Old: **2.47**
Delta	TotCoh	0.61 ± 0.03; n=21	0.63 ± 0.03; n=15	0.58 ± 0.04; n=18	F(12,306)=1.39 **P=0.17**	Adult vs. Young: **-0.14**; Old vs. Young: **0.14**; Adult vs. Old: **0.27**
Theta	TotCoh	0.63 ± 0.04; n=21	0.66 ± 0.03; n=15	0.63 ± 0.05; n=18	F(12,306)=1.39 **P=0.17**	Adult vs. Young: **-0.15**; Old vs. Young: **-0.01**; Adult vs. Old: **0.13**
Alpha 1	TotCoh	0.65 ± 0.04; n=21	0.67 ± 0.03; n=15	0.65 ± 0.05; n=18	F(12,306)=1.39 **P=0.17**	Adult vs. Young: **-0.10**; Old vs. Young: **0.03**; Adult vs. Old: **0.13**
Alpha 2	TotCoh	0.64 ± 0.04; n=21	0.66 ± 0.03; n=15	0.63 ± 0.05; n=18	F(12,306)=1.39 **P=0.17**	Adult vs. Young: **-0.08**; Old vs. Young: **0.04**; Adult vs. Old: **0.12**
Beta 1	TotCoh	0.64 ± 0.04; n=21	0.65 ± 0.03; n=15	0.64 ± 0.05; n=18	F(12,306)=1.39 **P=0.17**	Adult vs. Young: **-0.05**; Old vs. Young: **-0.01**; Adult vs. Old: **0.04**
Beta 2	TotCoh	0.65 ± 0.04; n=21	0.67 ± 0.03; n=15	0.67 ± 0.05; n=18	F(12,306)=1.39 **P=0.17**	Adult vs. Young: **-0.13**; Old vs. Young: **-0.09**; Adult vs. Old: **0.02**
Gamma	TotCoh	0.64 ± 0.04; n=21	0.67 ± 0.03; n=15	0.67 ± 0.05; n=18	F(12,306)=1.39 **P=0.17**	Adult vs. Young: **-0.16**; Old vs. Young: **-0.10**; Adult vs. Old: **0.04**

Table S1: Effect sizes were computed from the reported group means, standard errors of the mean (SEM), and sample sizes. Cohen's d was calculated using pooled standard deviation. For Golgi-Cox and immunofluorescence analyses, measurements were averaged per animal; the animal was treated as the biological replicate.

NOR

Comparison	Group 1 (G1)	Group 1 (G1)	Group 2 (G2)	Group 2 (G2)	Test	P value
	**Included**	**Excluded**	**Included**	**Excluded**		
Young (G1) vs. Adult (G2)	18	3	14	1	Fisher's exact test	0.6257
Young (G1) vs. Old (G2)	18	3	14	4	Fisher's exact test	0.6825
Adult (G1) vs. Old (G2)	14	1	14	4	Fisher's exact test	0.3457

OLT

Comparison	Group 1 (G1)	Group 1 (G1)	Group 2 (G2)	Group 2 (G2)	Test	P value
	**Included**	**Excluded**	**Included**	**Excluded**		
Young (G1) vs. Adult (G2)	12	0	9	2	Fisher's exact test	0.2174
Young (G1) vs. Old (G2)	12	0	13	2	Fisher's exact test	0.4872
Adult (G1) vs. Old (G2)	9	2	13	2	Fisher's exact test	>0.9999

Table S2: Exclusion-rate analyses for NOR and OLT tasks. Pairwise comparisons of the proportions of included versus excluded animals across experimental groups were performed using two-sided Fisher's exact test. No significant differences in exclusion rates were observed across any pairwise comparisons, indicating comparable exclusion distributions among groups.
